# DriverNet: uncovering the impact of somatic driver mutations on transcriptional networks in cancer

**DOI:** 10.1186/gb-2012-13-12-r124

**Published:** 2012-12-22

**Authors:** Ali Bashashati, Gholamreza Haffari, Jiarui Ding, Gavin Ha, Kenneth Lui, Jamie Rosner, David G Huntsman, Carlos Caldas, Samuel A Aparicio, Sohrab P Shah

**Affiliations:** 1 Department of Molecular Oncology, British Columbia Cancer Agency, 675 West 10th Avenue, Vancouver, BC, V5Z 1L3, Canada; 2 Faculty of Information Technology, Monash University, Wellington Road, Clayton, VIC 3800, Australia; 3 Department of Computer Science, University of British Columbia, 2366 Main Mall, Vancouver, BC, V6T 1Z4, Canada; 4 Bioinformatics Training Program, University of British Columbia, 570 West 7th Avenue, Vancouver, BC, V5Z 4S6, Canada; 5 Department of Pathology and Laboratory Medicine, University of British Columbia, 2211 Wesbrook Mall, Vancouver, BC, V6T 2B5, Canada; 6 Centre for Translational and Applied Genomics, BC Cancer Agency, 600 West 10th Avenue, Vancouver, BC, V5Z 4E6 Canada; 7 Cancer Research UK, Cambridge Research Institute, Li Ka Shing Centre, Robinson Way, Cambridge, CB2 0RE, UK

**Keywords:** driver mutations, sequencing, cancer, transcriptional networks.

## Abstract

Simultaneous interrogation of tumor genomes and transcriptomes is underway in unprecedented global efforts. Yet, despite the essential need to separate driver mutations modulating gene expression networks from transcriptionally inert passenger mutations, robust computational methods to ascertain the impact of individual mutations on transcriptional networks are underdeveloped. We introduce a novel computational framework, DriverNet, to identify likely driver mutations by virtue of their effect on mRNA expression networks. Application to four cancer datasets reveals the prevalence of rare candidate driver mutations associated with disrupted transcriptional networks and a simultaneous modulation of oncogenic and metabolic networks, induced by copy number co-modification of adjacent oncogenic and metabolic drivers. DriverNet is available on Bioconductor or at http://compbio.bccrc.ca/software/drivernet/.

## Background

Cancer genome sequencing experiments are designed to enumerate all somatic mutations within a cancer. Some of these mutations will serve as actionable genomic aberrations upon which to develop and apply targeted therapies (for example, mutations in *PIK3CA, BRAF*, and *KRAS*) and ultimately enabling rational frameworks for improved clinical management and patient care based on precise genomic patterns of somatic alteration. To this end, next generation sequencing (NGS) technology has shifted the rate-limiting step from identifying all cancer mutations in a sequenced genome to identifying the relatively few functional mutations that drive the phenotype of malignant cells. Therein lies a major challenge in the cancer genomics field: distinguishing pathogenic, driver mutations from the so-called passenger mutations that accrue stochastically, but do not confer selective advantages.

In order to discover novel driver mutations, several large-scale sequencing initiatives such as The Cancer Genome Atlas project (TCGA, for example, [1]) are generating simultaneous whole genome and transcriptome interrogations for hundreds of cases of the same tumor type. This opens the possibility of ascribing the impact of individual somatic mutations on gene expression networks. Initial observations in high-throughput datasets, coupled with innumerable functional studies suggest that driver mutations are expected to alter gene expression of their cognate proteins, their interacting partners, or genes that share the same biochemical pathway. This will lead to a correlated pattern of gene expression in a network of genes associated with a driver mutation, which differs from benign passenger mutations with little to no phenotype. Moreover, somatic aberrations in genes may alter more than one transcriptional network, thus enabling the enumeration of a group of pathways driven by a single genomic event. The importance of placing mutations in the context of their gene expression has been illuminated recently by Prahallad and colleagues [2], who established the therapeutic effect of PLX4032 against the *BRAF V600E *oncoprotein, which is mechanistically linked to the activation of *EGFR*. Thus, differential expression of *EGFR *in different cell types (colon cancers versus melanomas) has a dramatic impact on drug efficacy. Consequently, knowing active pathways coupled with mutational profiles will be critical for implementation of therapeutic decisions informed by the presence of mutations in a cancer.

Current approaches for driver analysis typically rely on the frequency of aberration of a given gene or locus in a population of tumors as a function of the background mutation rate (for example, [3-5]). Recent whole genome interrogations, however, have revealed the vast majority of mutated genes exhibit low population frequencies [6-10]. While most of these events can be explained by stochastically acquired mutations due to increased proliferation or acquisition of mutagenic processes, with no oncogenic properties, many others are in fact well-known pathogenic mutations with, in some cases, actionable clinical utility. For example, sequencing of complete exomes of 316 ovarian cancers [7] and 65 triple negative breast cancers [11] revealed rare but functionally important and actionable mutations (for example, in *ERBB2 *and *BRAF*) in a small percentage of cases that were not identified by frequency and background mutation rate analyses. Thus, frequency analysis will fail to recognize infrequent, but nonetheless important driver mutations.

**Figure 1:**
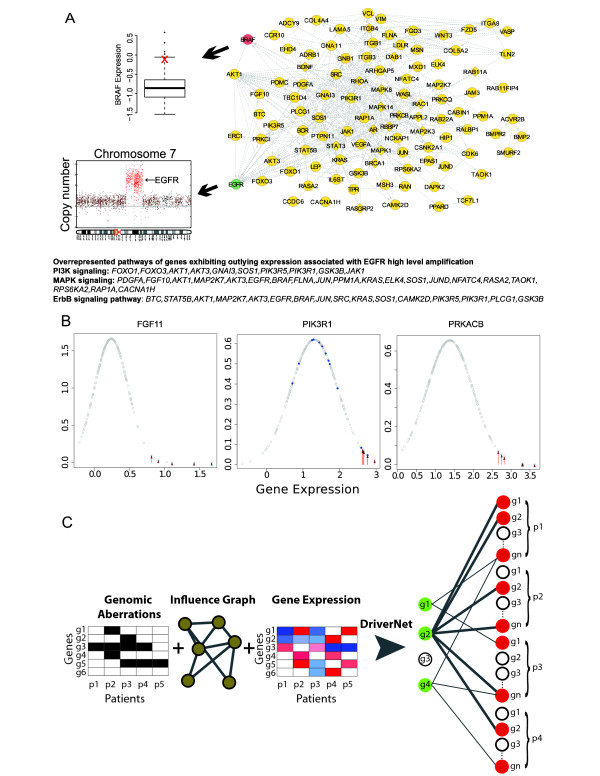
**A schematic showing how DriverNet works**. **(a) **An example of a Cytoscape visualization of a glioblastoma patient with a high-level amplification of epidermal growth factor receptor (*EGFR*) (shown in green) and coincident outlying expression of genes connected to EGFR in the Reactome influence graph (shown in yellow). Examples of the overrepresented pathways (by Reactome FI plug-in for Cytoscape, FDR < 0.001) from the list of genes showing outlying expression associated with the EGFR amplification are depicted at the bottom. The box plot shows the population-level expression distribution of BRAF, an interacting protein with *EGFR*, and where the specific case with EGFR amplification sits on that distribution (red 'x'). We note that in this case, *BRAF *itself is not mutated or amplified. **(b) **Fitted Gaussian expression distributions of three genes that interact with *EGFR*: *FGF11, PIK3R1*, and *PRKACB*, with each point indicating the probability density function for individual cases. For each gene, blue dots indicate cases with mutations in the gene itself and red arrows indicate cases with outlying expression with coincident *EGFR *amplifications. **(c) **Schematic representation of the DriverNet approach. Given the genomic aberration states for different patients and genes, gene expression data, and the influence graph, which captures biological pathway information, the bipartite graph shown on the right is constructed. Green nodes on the left partition of the bipartite graph correspond to aberrated genes and nodes on the right represent the outlying expression status for each patient where red indicates outlying patient-gene events from the gene expression matrix. The genes with the highest number of outlying expression events (for example, *g*2) are nominated as putative drivers.

We suggest that integrative analysis of genomic aberrations and transcriptional profiles in cancer will reveal somatic mutations that drive biological processes, regardless of the population frequency. Furthermore, we propose that biological networks can be leveraged to relate mutations to their consequent effect on transcription and gene expression. Figure 1A shows an example of high-level amplification of *EGFR *in a glioblastoma multiforme (GBM) tumor, accompanied by the coincident outlying expression of genes that are connected to *EGFR *through known biological pathways. We note that *BRAF *in this case, although not amplified itself, exhibits elevated expression compared to the population distribution. Other genes known to interact with *EGFR *exhibit similar extreme changes in expression levels in this example, such that PI3K signaling and MAPK signaling could be affected by this single genomic event. Figure 1B shows fitted Gaussian expression distributions of three genes that interact with *EGFR*: *FGF11, PIK3R1*, and *PRKACB*, and shows that some cases with outlying expression have coincident *EGFR *amplifications. Our assumption is that amplification of *EGFR *in these cases has driven expression of the example genes to the tails of their respective distributions. Thus, extreme changes in expression levels of genes related to genomic aberrations are observable in orthogonally measured high-throughput transcriptome assays. As such, simultaneous analysis of genome and transcriptome measurements should amplify important signals in the data. Motivated by this idea, we hypothesize that driver aberrations will measurably disrupt transcriptional profiles regardless of their frequency in the population.

Algorithmic frameworks to exploit the relationship between genomic events and consequent changes in gene expression to nominate putative driver genes are underdeveloped. We therefore propose an integrated genome/transcriptome analysis framework, called DriverNet, to contextualize genomic aberrations (for example, mutations and copy number alterations) by their effect on transcriptional networks and identify candidate genomic aberrations suitable for functional experimental follow-up. Our approach allows individual mutations to be related to coincident changes in gene expression and assigns statistical significance to candidate predictions, thus quantitatively and rationally prioritizing candidate genes. We note that our intent differs from complementary approaches such as the one described by Vaske *et al*. [12], which aims at nominating driver pathways rather than driver genes in cancer, and from those that leverage genome data without considering expression [4,13]. Both Masica and Karchin [14] and Ciriello *et al*. [15] integrate genome and transcriptome relationships in their framework; however, they differ from our approach, since Masica and Karchin [14] do not utilize known biological pathway information and Ciriello *et al*. [15] only consider mRNA expression associated with copy number aberrations and not with mutations. Other methods focusing on copy number and expression associations do not consider mutations, nor do they employ the use of previously annotated pathways [16,17].

To study the properties and advantages of our approach, we analyzed four large-scale genome-transcriptome interrogations of tumor populations (Table 1) in human gliomas, triple negative breast cancers, a population of nearly 1,000 breast tumors (all subtypes) and high-grade serous ovarian cancers. We present results from three experiments: i) ascertainment of sensitivity and specificity in the context of several cancer datasets; ii) enumeration of well-known, but infrequent, drivers modulating transcriptional networks, and iii) identification of complex driver events that implicate compound metabolic and oncogenic pathway modulation from single genomic events.

**Table 1:** Description of datasets

Dataset	Tumor type	Number of cases	Genomic aberrations	Outliers	Reference
GBM	glioblastoma	120	3,198	26,956	[6]
GBM2	glioblastoma	140	573	35,618	

METABRIC	breast	997	18,331	214,530	[19]

TN	triple negative breast	66	4,824	15,929	[11]
TN2	triple negative breast	66	1,019	15,929	

HGS	serous ovarian	304	8,229	91,697	[7]
HGS2	serous ovarian	307	4,919	92,491	

## Results

### Overview of DriverNet approach

We developed a novel, integrated algorithmic approach (DriverNet) to analyze population-based genomic and transcriptomic interrogations of tumor (sub)types for identification of pathogenic driver mutations. Our approach relates genomic aberrations to disrupted transcriptional patterns, informed by known associations or interactions between genes. The full details of the algorithm are described in the Online Methods, but will be summarized here in brief. Shown schematically in Figure 1C, DriverNet formulates associations between mutations and expression levels using a bipartite graph where nodes are: i) the set of genes representing the mutation status (the left partition of the graph) and ii) the set of genes representing outlying expression status in each of the patients (the right partition of the graph). For each patient, an edge between the nodes on the left and right partitions of the graph is drawn if the following three conditions are all satisfied: i) gene *g_i _*is mutated in patient *p *of the population (green nodes on the left partition of the graph); ii) gene *g_j _*shows outlying expression in patient *p *(red nodes on the right partition of the graph); and iii) *g_i _*and *g_j _*are known to interact according to pathway or gene set databases (an 'influence graph' after [18]). Our method then uses a greedy optimization approach to explain as many nodes on the right partition of the bipartite graph as possible using the fewest number of nodes on the left partition of the graph such that the genes explaining the highest number of outlying expression events (for example, *g*_2 _in Figure 1C) are nominated as putative driver genes. Finally, we apply statistical significance tests to these candidates based on null distributions informed by stochastic resampling.

### Datasets

For our analysis, we used four publicly available datasets that contain genome and transcriptome data of several tumor types (Table 1). Detailed descriptions of the analysis of the datasets and pre-processing workflows can be found in Additional file 1. The GBM dataset represents copy number, mutations and expression data for 120 glioblastoma multiforme patients [6] taken from the TCGA portal [19]. Note that the cases which had both mutation and copy number data were included in this dataset. The METABRIC dataset [20] represents copy number alterations and accompanying gene expression data for 997 breast cancer patients. TN represents the validated mutations, copy number, and expression data for 66 triple negative breast cancer patients [11]. The TCGA HGS dataset contains mutations, copy number, and expression data for 304 high-grade serous ovarian cancer patients [7] that were taken from the TCGA portal. Like the GBM dataset, we only included the cases which had both mutation and copy number data. The data analysis workflow is shown schematically in Additional file 2. The GBM2, TN2, and HGS2 datasets represent mutations only and gene expression data for 140, 66, and 307 glioblastoma, triple negative, and high-grade serous ovarian cancer patients, respectively.

### Performance benchmarking analysis establishes DriverNet as a sensitive and specific algorithm

In practice, quantitative measurements with standard sensitivity/specificity benchmarking techniques are impractical in the absence of ground truth. However, due to the availability of well-studied cancer gene databases, including the cancer gene census (CGC) [21] and the catalogue of somatic mutations in cancer datasets (COSMIC) [22], we set out to approximate performance metrics and compare DriverNet with the following two competing methods: i) a method described by Masica and Karchin [14], which uses correlation-based statistics followed by a Fisher exact test to associate mutations with gene expression patterns (referred to as 'Fisher', see Additional file 1), ii) a method described in Youn and Simon [5], which identifies driver genes based on the background mutation rate, functional impact on proteins, and redundancy in genetic code (referred to as 'Frequency'). In adherence to both approaches mentioned above, we removed copy number data from the analysis and restricted the comparisons to mutation data only (GBM2, TN2, and HGS2, Table 1), resulting in the exclusion of the METABRIC dataset as it contained copy number aberration data only. We used two systematic benchmarking measures as follows: i) examining the proportion of predictions found in the Cancer Gene Census (CGC) database [21]; ii) examining the prevalence of somatic mutations of candidate genes in accordance with the COSMIC database, assuming genes with higher mutation prevalence in the corresponding patient population of interest in COSMIC (glioblastoma, breast and ovarian cancer) are more likely to be driver genes. Theoretically, this measure should favor the Frequency approach.

**Figure 2:**
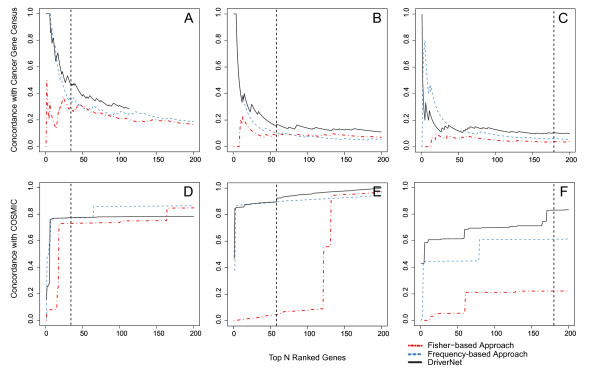
**DriverNet performance benchmarking with the GBM2, HGS2, and HGS2 datasets**. **(A-C) **Concordance with Cancer Gene Census for DriverNet, Frequency-based, and Fisher-based approaches as a function of the top *N *ranked genes (out of 200) for the GBM2, TN2, and HGS2 datasets, respectively. **(D-F) **Concordance with the COSMIC database (cumulative distribution of mutation prevalence in the COSMIC database) for DriverNet, Frequency-based, and Fisher-based approaches as a function of the top *N *ranked genes (out of 200) for the GBM2, TN2, and HGS2 datasets, respectively. Note that for the GBM2 dataset, DriverNet nominates 113 genes as candidate drivers, therefore, the concordance of DriverNet genes with the Cancer Gene Census is plotted for the 113 candidates.

To systematically evaluate specificity, we compared the proportion of predictions that were present in CGC as a function of decreasing sensitivity thresholds (Figure 2A, B, C) for all three methods. We also looked at the cumulative distribution of mutation prevalence in the COSMIC database for all three datasets (Figure 2D, E, F). Throughout the range of the top predictions output by DriverNet, the concordance with CGC was always higher than for Fisher and Frequency in the GBM2 and TN2 datasets. For HGS2, DriverNet and the Frequency approach outperformed the Fisher method. The cumulative prevalence in the COSMIC dataset was higher for DriverNet compared to the other two approaches throughout the range of the top predictions, with Frequency second best. Thus, far fewer predictions are required by DriverNet to capture the majority of drivers in the dataset, indicating higher relative specificity.

For GBM2 (mutations only), the Frequency method identified eight genes: *EGFR, IDH1, NF1, PIK3R1, PTEN, RB1, TP53*, and *FKBP9 *as significantly altered with seven of these found in CGC (Additional file 3). In total, DriverNet identified 34 genes (*p *< 0.05) including seven of the genes nominated by the Frequency-based approach (Additional file 4). Several genes found in CGC (*PIK3C2G, MDM2, BCR, ERBB2, DDIT3, FGFR1, BRCA2, MET*, and *PDGFRA*) were also among the top 34 genes nominated by DriverNet. We detected *MET *as the 29th ranked gene (*p *= 0.002, mutated in three cases), which was reported in [1], suggesting that it has been overlooked by the Frequency method, which ranked this gene as the 93rd.

For TN2 (mutation only, no copy number), the Frequency method identified five genes: *PIK3CA, RB1, TP53, PTEN*, and *MYO3A *as significantly altered genes by mutation, of which four were found in CGC (Additional file 5). In total, DriverNet identified 59 genes with *p *< 0.05, four of which were nominated by the Frequency-based approach (Additional file 6). A DriverNet prediction not identified by the Frequency approach included *JAK1 *(*p *= 0, ranked 13th, mutated in one case), which plays a key role in prolactin signaling, which is implicated in breast cancer [23,24].

For HGS2 (mutation only, no copy number), the Frequency method identified *CSMD3, BRCA1, BRCA2*, and *TP53 *as significantly altered genes, three of which were found in CGC (Additional file 7). DriverNet identified *BRCA1, BRCA2*, and *TP53 *in addition to CGC genes, *KRAS, PTEN, KIT, NRAS, RPN1, RB1, PIK3CA, CLTCL1, ATIC, CREBBP, MET, PPP2R1A, CLTC, CTNNB1, BRAF*, and *TSHR *(Additional file 8). *BRAF, PIK3CA, KRAS*, and *NRAS *are known oncogenic drivers and emphasize the power of integration of expression data to nominate important but infrequently mutated genes. In addition, the known tumor suppressor gene, *PTEN*, was among the top genes in DriverNet (rank 11th) but was overlooked by the Frequency method, which ranked this gene as 525th.

### Infrequent mutations modulating transcriptional networks feature prominently in population level datasets

We then sought to ascertain the prevalence of rare drivers in all four datasets overlooked by Frequency-based approach to driver prediction. We identified 'infrequent' significant drivers (*p *< 0.05) where the gene of interest was abrogated by mutation or copy number alteration (CNA) in < 2% of cases. Due to unknown ground truth with respect to actual drivers, we restrict presentation to those genes also found in the CGC. This resulted in 22 genes in METABRIC, 13 genes in HGS, 1 gene in TN, and 2 genes in GBM (Table 2). The infrequent drivers in METABRIC were *PTEN, RB1, MDM2, MYC, CDKN2A, CLTC, CREBBP, GNAS, EGFR, CCNE1, EP300, CBL, PIK3R1, JAK2, TP53, NUP98, PIK3CA, IDH2, KRAS*, and *TRA*@. Both *PIK3CA *(two cases with high-level amplifications) and *PIK3R1 *(two cases with homozygous deletions) were altered in 0.19% of cases, and yet showed evidence of driving expression levels of the connected genes to the tails of the expression distribution. Interestingly, we identified seven cases (0.67%) with homozygous deletions in *TP53 *(locus 17p13.1) coincident with outlying expression in MAPK and Wnt signaling pathways (Additional files 9 and 10). Loss of function of *TP53 *is typically associated with mutation; however, these results suggest that in rare cases, homozygous deletions may be the mechanism by which *TP53 *is lost in breast cancer.

**Table 2:** The predicted rare drivers

Dataset	Gene	Gband	SNV/Indel	HLAMP	HOMD	Corrected *P *value	Percent altered
METABRIC	*PTEN*	10q23.31	0	0	16	0	1.54
METABRIC	*RB1*	13q14.2	0	0	16	0	1.54
METABRIC	*MDM2*	12q15	0	11	0	0	1.06
METABRIC	*MYC*	8q24.21	0	10	0	0	0.96
METABRIC	*CDKN2A*	9p21.3	0	0	16	0	1.54
METABRIC	*CLTC*	17q23.1	0	16	0	0	1.54
METABRIC	*CREBBP*	16p13.3	0	1	2	0	0.29
METABRIC	*GNAS*	20q13.32	0	7	0	0	0.67
METABRIC	*EGFR*	7p11.2	0	3	1	0	0.39
METABRIC	*CDH1*	16q22.1	0	0	16	0	1.54
METABRIC	*CCNE1*	19q12	0	6	1	0	0.67
METABRIC	*EP300*	22q13.2	0	0	4	0	0.39
METABRIC	*CBL*	11q23.3	0	0	13	0	1.25
METABRIC	*PIK3R1*	5q13.1	0	0	2	1.00E-04	0.19
METABRIC	*JAK2*	9p24.1	0	0	7	1.00E-04	0.67
METABRIC	*TP53*	17p13.1	0	0	7	2.00E-04	0.67
METABRIC	*NUP98*	11p15.4	0	0	8	0.0011	0.77
METABRIC	*ATM*	11q22.3	0	0	15	0.0149	1.45
METABRIC	*PIK3CA*	3q26.32	0	2	0	0.017	0.19
METABRIC	*IDH2*	15q26.1	0	4	1	0.017	0.48
METABRIC	*KRAS*	12p12.1	0	3	1	0.0348	0.39
METABRIC	*TRA@*	14q11.2	0	1	5	0.0388	0.58

TN	*JAK1*	1p31.3	1	0	0	0.0026	1.5

HGS	*AKT2*	19q13.2	0	3	1	0	1.32
HGS	*KIT*	4q12	5	0	1	2.00E-04	1.97
HGS	*NRAS*	1p13.2	2	0	0	9.00E-04	0.66
HGS	*RPN1*	3q21.3	2	0	0	0.0019	0.66
HGS	*PIK3CA*	3q26.32	2	0	0	0.0029	0.66
HGS	*CREBBP*	16p13.3	5	0	1	0.0031	1.97
HGS	*PPP2R1A*	19q13.33	3	0	1	0.0046	1.32
HGS	*ATIC*	2q35	2	0	1	0.005	0.99
HGS	*CLTCL1*	22q11.21	4	0	1	0.0068	1.64
HGS	*MET*	7q31.2	4	0	0	0.0132	1.32
HGS	*MAP2K4*	17p12	1	0	2	0.044	0.99
HGS	*ETV1*	7p21.2	1	1	1	0.0468	0.99
HGS	*EP300*	22q13.2	1	0	3	0.0492	1.32

GBM	*KRAS*	12p12.1	1	0	1	1.41	1.67
GBM	*AKT1*	14q32.33	0	1	0	1.64	0.83

In HGS, we found 13 genes that were infrequent drivers also found in CGC (*AKT2, KIT, NRAS, RPN, PIK3CA, CREBBP, PPP2R1A, ATIC, CLTCL1, MET, MAP2K4, ETV1*, and *EP300*) (Table 2). Intriguingly, *KIT *(1.97% of cases) and *NRAS *(0.66% of cases) were detected as drivers (*p *= 2E-4 and 9E-4, respectively; Additional files 11 and 12) where *KIT *is mutated in melanomas, gastrointestinal stromal tumors, adult acute myeloid leukemia patients, and many other tumor types at high frequency and is the target of the kinase inhibitor Imatinib. The mutations in *NRAS *(typically associated with melanomas, multiple myelomas, acute myelogenous leukemia, and thyroid cancer) were, in both cases, the Q61R hotspot mutation in the Ras domain. Both the *KIT *and *NRAS *mutations were overlooked as driver mutations by the Frequency-based approach (Additional file 7). This illustrates the increased sensitivity of DriverNet in identifying infrequent drivers in the population. Interestingly, mutations typically associated with lower grade (Type I) ovarian cancers such as *PIK3CA *(0.66% cases mutated) and *CTNNB1 *(0.6% cases mutated) were also nominated as drivers despite having extremely low frequency. The two *PIK3CA *mutations were both in well-known, activating hotspots, E545K and H1047R. We suggest that these (four separate) cases might actually be histologically misdiagnosed ovarian cancers. These cases represent an important anecdote as many tumor populations contain rare mutations that create aberrant expression profiles. Type I ovarian cancers exhibit considerably different expression profiles compared to Type II high-grade serous cancers [25]. If indeed these cases are non-serous it would be unsurprising, given the DriverNet formulation of integration of genomic and transcriptomic profiles, that these rare mutations would cover many outlier events. In addition, we note that the previously mentioned *MAP2K4 *as an infrequent driver with a mutation in one case and homozygous deletions in two cases, and the presence of *ETV1*, typically known for gene fusions, are listed amongst the infrequent drivers in the HGS ovarian data. Finally, we cross-referenced the list of genes *p *< 0.05 with Cheung *et al*. [26] (a list of genes with genetic vulnerabilities in cancer cell lines) and noted that *ALG8 *and *CCNE1 *overlapped.

In the TN and GBM datasets, results were sparser. In the TN dataset, only one gene was an infrequent driver that was also in CGC: *JAK1 *with a mutation occurring in a single case (Table 2). *JAK1 *associated outliers were enriched for EGFR1 signaling (Additional files 13 and 14), suggesting that the mutation has downstream effects on an important oncogenic signaling network. In the GBM dataset, two genes, namely *KRAS *and *AKT1*, were infrequent drivers and were also found in CGC. *KRAS *associated outliers were enriched for MAPK and PDGFR signaling and *AKT1 *outliers were enriched for FoxO family signaling (Additional files 15 and 16). AKT activation is associated with many malignancies, where AKT acts, in part, by inhibiting FoxO tumor suppressors [27]. Collectively, investigations of rare drivers in METABRIC, HGS, TN, and GBM point out *bona fide*, but rare driver mutations, which would likely be omitted by methods examining genomic aberrations by selection or frequency analysis. These results indicate that rare driver mutations modulating expression networks comprise a meaningful component of the landscape of transcriptional variation attributed to the somatic genome, and thus should not be overlooked in the comprehensive enumeration of driver mutations in population-level studies.

### Genomic copy number changes harboring known oncogenes simultaneously modulate metabolic pathways

**Figure 3:**
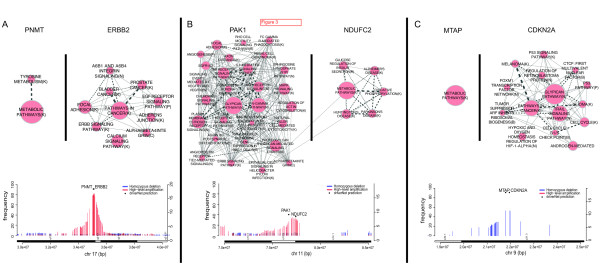
**Simultaneous modulation of metabolic pathways in copy number alterations harboring known oncogenes**. EnrichmentMap [32] diagrams depicting Reactome pathways enriched in the set of outliers associated with pairs of genes that are co-amplified or co-deleted. In each pair, one gene is a known tumor suppressor or oncogene while the other is a metabolism gene. Pathways are shown as connected nodes in a graph where the size of the node indicates the number of genes in the pathway. Edges between nodes indicate genes common to both pathways where the thickness of the edge represents the degree of overlap. In general, little overlap was observed between metabolic drivers and oncogenic/tumor-suppressor drivers. **(A) ***PNMT *and *ERBB2 *co-amplified genes at the chr17q12 locus in breast cancer. **(B) ***PAK1 *and *NDUFC2 *co-amplified genes at the 11q14 locus in breast cancer. **(C) ***CDKN2A *and *MTAP *co-deleted genes at chr9p21.3 in GBM.

We next examined patterns of modulated expression associated with drivers occurring within the same high-level amplification or homozygous deletion. Surprisingly, we noted four examples in the METABRIC and GBM datasets whereby genes proximal to known drivers and within the same genomic copy number change exhibited evidence for altering the expression of metabolic pathways exclusive of known oncogenic or tumor suppressor pathway modulation (Figure 3). *PNMT *encodes the phenylethanolamine N-methyltransferase enzyme and resides approximately 20 Kb centromeric to *ERBB2 *with one intervening gene. *ERBB2*, amplified in approximately 15-20% of breast cancers, is a well-known, targetable membrane-bound growth-factor receptor that is effectively inhibited by trastuzumab in clinical practice. The proximity of *PNMT *to *ERBB2 *results in co-amplification of both genes in nearly all cases (82/83 cases with high-level amplification of *ERBB2 *(Additional file 10)). *PNMT *was the top ranked driver in our analysis (*ERBB2 *was rank 3). When we examined the outlier genes associated with *ERBB2 *and *PNMT, ERBB2*-associated outlier genes were, as expected, enriched for Erbb signaling and EGF signaling pathways. *PNMT*-associated outliers were enriched for non-oncogenic macromolecule biosynthesis pathways including metabolic pathways and tyrosine metabolism (Figure 3A). The co-occurring modulation of oncogenic and metabolic pathways was also found in other high-level amplifications in METABRIC including the 11q14 amplification of *PAK1 *and *NDUFC2 *(Additional file 10). *PAK1 *(27 cases with high-level amplifications) shows evidence of driving *EGFR *signaling (Figure 3B) and importantly segregates with a poor outcome ER positive subtype as reported in [20]. *NDUFC2 *(30 cases with high-level amplifications), downstream of *PAK1 *by approximately 660 Kb, encodes an NADH dehydrogenase enzyme. Outliers associated with *NDUFC2 *were associated with metabolic pathways and an oxidative phosphorylation pathway: a metabolic pathway that uses energy released by the oxidation of nutrients to produce adenosine triphosphate (Figure 3B).

A similar pattern of simultaneous modulation of metabolic pathways by the copy number changes harboring known oncogenes was observed in GBM data. The cyclin-dependent kinase *CDKN2A *and the methylthioadenosine phosphorylase *MTAP *are separated by approximately 100 Kb and are adjacent genes. *MTAP *(DriverNet rank 3) and known tumor-suppressor *CDKN2A *(DriverNet rank 4) are known to be co-deleted and they were observed as such in our analysis. We observed 53 cases with homozygous deletions in *CDK2NA *with accompanying co-deletion of *MTAP *in all cases (Additional file 16). In two additional cases with *CDKN2A *point mutations, *MTAP *was not found to be mutated or deleted. The enriched pathways of the *CDK2NA*-associated outliers included cell cycle, p53 signaling, and the FOXM1 transcription factor network amongst others. The only significant enriched pathway of MTAP-deletion associated outliers was the metabolic pathway (Figure 3C).

We examined *PNMT*-, *NDUFC2*-, and *MTAP*-associated outlying genes that were part of metabolic pathways and also *ERBB2*-, *PAK1*-, and *CDKN2A*-associated outlying genes that were related to the oncogenic/tumor suppressor pathways. Outlying genes related to metabolic pathways and oncogenic/tumor suppressor pathways were distributed across disparate loci in the genome eliminating co-amplification as the cause for the observed signals (Additional file 17).

The results of metabolic genes being co-aberrated with oncogenic and tumor suppressor genes suggest strongly that at least a portion of metabolic pathway disruption in cancer can be mechanistically attributed to somatic aberrations in the genome. Moreover, our results indicate the intriguing possibility that genomic aberrations harboring known oncogenic/tumor suppressor drivers are being selected for due to oncogenic pathway modulation coupled with non-overlapping metabolic pathway modulation.

## Discussion

A major challenge in large-scale interrogation of genomic and transcriptomic profiles of tumor types is to contextualize genomic aberrations within their gene expression profiles. Assessing the impact of a somatic mutation on the expression networks of a tumor provides strong evidence for its status as a driver. We presented a novel algorithm called DriverNet for integrative analysis of genomic and transcriptomic data derived from population-level studies of tumors. DriverNet associates the presence of a mutated gene with its impact on the gene expression levels of its known interacting partners. We showed in several cancer datasets that this approach is both sensitive and specific with respect to known driver genes and is suitable for application in population-level datasets for numerous tumor types that will rapidly emerge in the coming years.

Investigation of infrequent drivers revealed a surprising number of rare mutations in known cancer genes typically associated with other cancers. Although infrequent, they nonetheless modulate the expression profiles and their identification is critical to understanding the pathogenesis of the cancers that harbor them. We suggest that examination of genomic patterns in the population without the integration of the transcriptome would likely result in overlooking these important, but rare drivers. The structure of the bipartite graph induces an interplay between the influence graph, the frequency of mutations, and the frequency of aberrant expression. A natural question that arises is the role of both frequency of mutation and node degree in the ranking of the output. Additional files 18 and 19 show that while rank is correlated with both frequency and node degree, the relationship is not monotonic and therefore the structure of the graph does not deterministically order the output. This suggests instead that simultaneous observations in the genome and the transcriptome in many cases override the structure induced by the influence graph and mutation frequency and can therefore penetrate the seemingly deterministic structure induced by the initial bipartite graph.

Finally, we describe a set of aberrations whereby proximal drivers appear to simultaneously modulate oncogenic and metabolic pathways. This was observed in both breast cancer and GBM datasets and leaves open the possibility that selection of well-known drivers such as *ERBB2 *and *EGFR *may be synergistically acting on altered metabolic processes abrogated by co-altered, nearby metabolism genes. In light of recent renewed interest in studying altered metabolism in cancer [28] owing to IDH1/2 somatic mutations in AML and GBM, the compound effects of single genomic events on metabolic and oncogenic pathways, suggest that disruption of metabolic pathways by somatic mutations may be more widespread than previously thought and provides an impetus for novel therapies that might restore normal metabolic function in a cancer-cell specific manner.

### Limitations

The DriverNet algorithm has some limitations. As outlying expression is computed in a deterministic manner, we may not be capturing less extreme but nonetheless important changes in expression that are modulated by a genomic event. Furthermore, DriverNet does not gracefully handle the directionality of the expression change. A probabilistic model would account for the subtler changes in expression handling; however, the combinatorial complexity of inference required in a fully probabilistic framework remains a daunting and unresolved challenge because of the number of parameters to estimate. Thus, this remains an open problem. In addition, DriverNet relies on the genomic aberrations including mutations and extreme copy number alteration events that are supplied to the algorithm. The threshold to determine what constitutes a significant copy number alteration lies within third-party copy number analysis algorithms and can affect DriverNet results. Performance benchmarking suggest that, in most cases, DriverNet performs better when only extreme copy number alterations, that is, high-level amplifications and homozygous deletions, were included in the analysis (Additional file 20). Reducing the thresholds to detect more copy number alterations (such as chromosome-arm level events) results in too large a space of altered genes in a given dataset (Additional files 21, 22, 23, 24).

The DriverNet framework relies on a predetermined influence graph that is undoubtedly sparse and incomplete. This is underscored by the omission in the METABRIC dataset of *ZNF703*, which resides in the amplification of the 8p12 locus that includes *FGFR1*. We have recently described *ZNF703 *as a driver [29] in luminal B cancers; however, DriverNet was not positioned to identify it due to its absence in the Reactome database. There are undoubtedly other false negative predictions due to poor characterization and lack of protein-protein interaction data; however, as interaction databases increase in density and volume of interactions, the DriverNet framework will be well placed to leverage such improvements. Nevertheless, our goal is not to discover new protein interactions in this work, but rather to describe the association of mutations and expression in the context of well-understood knowledge bases. Finally, we note that this framework is suitable for datasets with many patients sequenced. Ultimately, we wish to extend the framework for application to individual patients to determine the effectiveness of identification of actionable driver mutations for clinical use. This will require the accumulation of large gene expression repositories for tumor types that can be used to contextualize a patient's expression and mutational profiles.

## Conclusions

We have presented a comprehensive analysis from four independent datasets of how transcriptional networks are affected by genomic aberrations in cancer and demonstrate how integrative analysis can be used effectively to identify novel driver genes in population-level studies of tumor genomes and transcriptomes. Our results demonstrate the power of integrative analysis across multiple tumor types in recently generated population-scale datasets in revealing infrequent, but functionally important, mutations and novel patterns of pathway disruption in cancer. We expect DriverNet to generalize well to planned future studies, including application to patient-specific mutational and expression profiles for genome/transcriptome-informed personalized cancer care.

## Methods

In this section we present the essential details of the DriverNet algorithm. Additional details of data analysis, data preprocessing, and the Fisher method are presented in Additional file 1.

### Details of DriverNet algorithm

Consider two gene-patient matrices. The first matrix *M*(*i, j*) represents a binary matrix where *M*(*i, j*) = 1 indicates gene *i *is mutated in patient *j *and *M*(*i, j*) = 0 indicates the absence of a mutation. Mutations can take the form of somatic point mutations, indels, copy number changes, or possibly epigenomic events. Matrix *G*(*i, j*) captures the real-valued gene expression measure of gene *i *in patient *j *and can be derived from gene expression arrays or RNASeq. Optionally, *G*(*i, j*) can be transformed into a matrix *G'*(*i, j*) indicating whether gene *i *in patient *j *is an outlier from the population-level distribution for that gene. Given these matrices, we can formulate the problem of finding driver mutations with a bipartite graph,  (Figure 1C), where nodes on the left represent genomic aberration status from *M *(green nodes show the genes that have a mutation in at least one patient) and nodes on the right are patient-gene events from *G *or *G*' (for every patient, outliers are shown as red nodes). Edges are drawn between nodes in different partitions of the graph under the following conditions: for each patient *p_k _*draw an edge between nodes *g_i _*in the left partition and *g_j _*for patient *p_k _*in the right partition, if *g_i _*is mutated, *g_j _*exhibits outlying expression, and *g_i _*and *g_j _*interact according to known gene networks (for example, Reactome FI [30]), termed the *influence graph *after [18].

The aim of the inference algorithm is to identify genes in the left partition that are connected to the most nodes in the right partition (for example, *g2 *as shown in Figure 1C), thereby identifying mutated genes with the largest extent of transcriptional disruption, and simultaneously implicating a network of connected genes in the influence graph with outlying expression that associate with the mutation. The genes are ranked according to their node coverage in the bipartite graph, . If we denote the set of all the mutated genes by *U*, we postulate that the top *n *driver geneset *D_n _*⊆ *U *is the set of *n *genes that cover the maximum number of nodes on the right partition of the bipartite graph. It should be noted that: i) due to different factors, all the outlying expression events may not be explained by the given mutations; and ii) the algorithm formulation makes the strong assumption that drivers will modulate the expression of many genes, which will primarily apply for genes that alter large, well-defined transcriptional networks. Finally, we observe that solving this problem is closely related to the minimum set cover problem, which is NP-hard.

### A greedy approximation algorithm to solve the optimization problem

Given a set of elements (called the universe) and some sets whose union comprises the universe, the set cover problem is to identify the smallest number of sets whose union still contains all elements in the universe. The analogy of the minimum set cover problem to our driver mutation framework is as follows: i) elements of the universe are the patient-gene (outlying expression) events, and ii) each mutation corresponds to a set that consists of those patient-gene events connected to this mutation in the bipartite graph. The greedy algorithm for our problem is similar to that for the set cover problem: at each stage, choose a mutated gene that contains the largest number of uncovered outlying expression events (see Algorithm 1). The stopping condition is when all the connected outlying expression events are covered. In other words, the algorithm looks for the minimum covering for all of the elements in the universe. It can be shown that the greedy algorithm achieves an approximation ratio of *H*(*s*), where *s *is the size of the largest set and $H\left(n\right)=\sum_{k=1}^{n}1/k$ is the *n*th harmonic number.

**Algorithm 1:** Greedy driver gene selection algorithm

Require: be the bipartite graph, where denotes the set of nodes corresponding to mutated genes, denotes the set of nodes corresponding to the patient-specific outlying expression events, and denotes the set of edges between and	
1:	//the set of selected driver genes
2:	//the number of all the connected outlying expression events
3: *z *← 0	//the number of covered outlying expression events so far
4: while *z *< Z do	
5:	//pick mutated gene with the highest degree; in case of a tie, randomly pick one of the genes
6:	//update the number of covered outlying events
7:	//add *g *to the driver set
8:	
9: for *g*' ∈ *S *do	
10:	//remove the node *g' *and its connected edges from
11: end for	
12: end while	
13:	

### Significance tests

The statistical significance of the driver genes are assessed using a randomization framework. The original datasets are permuted *N *= 500 times, and the algorithm is run on the *N *randomly generated datasets and results on real data are assessed to see if they are significantly different from the results on randomized datasets. This is an indirect way of perturbing the bipartite graph corresponding to the original problem. To generate the random datasets, we permute both the patient-mutation, *M *, and patient-outlier, *G*', matrices according to the following procedure: i) construct a *J *× *K *zero matrix where *J *represents the number of patients and *K *represents the total number of Ensmbl 54 protein-coding genes, ii) put 1 in *N*_total _randomly selected cells, where *N*_total _represents either the total number of mutations or the total number of outlying genes depending on which matrix is permuted, iii) remove the columns where their elements are 0. Using the same influence graph, the algorithm is run on the *N *= 500 permuted patient-mutation, *M*_1_... *M_N_*, and patient-outlier, *G*_1_'... *G_N_*', matrices.

Suppose *D *is the result of the driver mutation discovery algorithm. *D *contains a ranked list of driver genes with their corresponding node coverage in the bipartite graph, . The statistical significance of a gene *g *∈*D *with a corresponding node coverage, COV*_g_*, is the fraction of times that we observe driver genes with the node coverage of more than COV*_g _*in the *N *= 500 random runs of the algorithm:

$$\text{pvalue}\left(g\right)=\frac{\overset{N}{\underset{i=1}{\sum }}\overset{S_{i}}{\underset{j=1}{\sum }}\delta \left[\text{CO}{\text{V}}_{gij}>\text{CO}{\text{V}}_{g}\right]}{\overset{N}{\underset{i=1}{\sum }}S_{i}}$$

where *S_i _*is the number of drivers identified in the *i*th run of the algorithm. We then use the Benjamini-Hochberg approach for correcting the *P *values for multiple tests.

### Building the influence graph

The influence graph captures the knowledge about the influence of mutation in a gene on the change of expression of another gene. Various sources of information such as the protein-protein interaction (PPI) networks or networks based on copy number and/or expression data can be used to build the influence graph. In this paper, we utilize the protein functional interaction network derived in [30] to build the influence graph. This network extends the protein functional interaction network in curated pathways with non-curated sources of information, including protein-protein interactions, gene co-expression, protein domain interaction, gene ontology (GO) annotations, and text-mined protein interactions, which cover close to 50% of the human proteome.

### Implementation

The DriverNet algorithm is implemented in a publicly available R package [31]. The memory complexity of the greedy algorithm is *O*(*M N *+ *M R *+ *R*^2)^, where *M *is the number of patients, *N *is the number of mutated genes, and *R *is the number of genes with gene expression values and also in the influence graph. The algorithm needs memory to hold the patient-mutation matrix, the patient-outlier matrix, and the influence graph. Note that all the three matrices are sparse binary matrices, thus the memory usage can be decreased by using sparse representation of the matrices. If we rank all the mutated genes, the time complexity is O(δ × *N *(*N *+ 1)/2), where δ is the time used to compute the explained outliers by a gene, which is bounded by its node degree of the influence graph. In practice, the algorithm is fast when the memory usage is low. For example, for the GBM dataset, it takes about 1 minute to run on a dual-core desktop Mac computer without computing the empirical *P *values.

## Abbreviations

AMP: amplifications; CGC: cancer gene census; CNA: copy number alteration; COSMIC: catalogue of somatic mutations in cancer datasets; GBM: glioblastoma multiforme; GO: gene ontology; HETD: hemizygous deletion; HLAMP: high-level amplification; HOMD: homozygous deletion; MTAP: methylthioadenosine phosphorylase; NGS: next generation sequencing; PPI: protein-protein interaction; TCGA: the cancer genome atlas.

## Authors' contributions

SS was responsible for the project's conception and oversight. GH, AB, and SS designed and/or implemented different parts of the research plan and wrote the manuscript. AB, GH, JD, GaH, and JR conducted the analyses of the data. KL, GH, AB, and JD contributed to the R package. CC and SA are METABRIC project leaders. SA is TN sequencing project leader. SA and DH contributed to the project's conception. All authors read and approved the final manuscript.

## Supplementary Material

Additional file 1 **Supplementary text**. Click here for file

Additional file 2 **Data analysis workflow**. Click here for file

Additional file 3 **Ranked list of candidate driver genes using the Youn-Simon approach for the GBM2 dataset**. rank: rank of the gene, hgnc_symbol: gene symbol, p.value: *P *value, p.adjust: adjusted *P *value using the Benjamini-Hochberg approach. Click here for file

Additional file 4 **Ranked list of candidate driver genes for the GBM2 dataset**. rank: rank of the gene according to DriverNet, gene: gene symbol, gband: gene chromosome location and gene band, SNV.Indel: number of cases with SNV or indel in that specific gene, HLAMP: number of cases with copy number high-level amplifications, AMP: number of cases with copy number amplifications, HOMD: number of cases with copy number homozygous deletions, HETD: number of cases with copy number hemizygous deletions, covered events: the number of events (edges) connected to the gene on the left of the bipartite graph, node degree: the number of genes connected to the gene of interest in the influence graph, p.value: *P *value corrected for the multiple test using the Benjamini-Hochberg approach, CGC.status: Cancer Gene Census (CGC) membership status (1 = found in CGC, 0 = not in CGC), percentage.event: percentage of cases with genomic aberrations in the gene of interest, p.way: top pathways associated with outlying genes (posterior probability > 0.8); numbers in parentheses show the posterior probability. Click here for file

Additional file 5 **Ranked list of candidate driver genes using the Youn-Simon approach for the TN2 dataset**. rank: rank of the gene, hgnc_symbol: gene symbol, p.value: *P *value, p.adjust.BH: adjusted *P *value using the Benjamini- Hochberg approach. Click here for file

Additional file 6 **Ranked list of candidate driver genes for the TN2 dataset**. rank: rank of the gene according to DriverNet, gene: gene symbol, gband: gene chromosome location and gene band, SNV.Indel: number of cases with SNV or indel in that specific gene, HLAMP: number of cases with copy number high-level amplifications, AMP: number of cases with copy number amplifications, HOMD: number of cases with copy number homozygous deletions, HETD: number of cases with copy number hemizygous deletions, covered events: the number of events (edges) connected to the gene on the left of the bipartite graph, node degree: the number of genes connected to the gene of interest in the influence graph, p.value: *P *value corrected for the multiple test using the Benjamini-Hochberg approach, CGC.status: Cancer Gene Census (CGC) membership status (1 = found in CGC, 0 = not in CGC), percentage.event: percentage of cases with genomic aberrations in the gene of interest, p.way: top pathways associated with outlying genes (posterior probability > 0.8); numbers in parentheses show the posterior probability. Click here for file

Additional file 7 **Ranked list of candidate driver genes using the Youn-Simon approach for the HGS2 dataset**. rank: rank of the gene, hgnc_symbol: gene symbol, p.value: *P *value, p.adjust: adjusted *P *value using the Benjamini-Hochberg approach. Click here for file

Additional file 8 **Ranked list of candidate driver genes for the HGS2 dataset**. rank: rank of the gene according to DriverNet, gene: gene symbol, gband: gene chromosome location and gene band, SNV.Indel: number of cases with SNV or indel in that specific gene, HLAMP: number of cases with copy number high-level amplifications, AMP: number of cases with copy number amplifications, HOMD: number of cases with copy number homozygous deletions, HETD: number of cases with copy number hemizygous deletions, covered events: the number of events (edges) connected to the gene on the left of the bipartite graph, node degree: the number of genes connected to the gene of interest in the influence graph, p.value: *P *value corrected for the multiple test using the Benjamini-Hochberg approach, CGC.status: Cancer Gene Census (CGC) membership status (1 = found in CGC, 0 = not in CGC), percentage.event: percentage of cases with genomic aberrations in the gene of interest, p.way: top pathways associated with outlying genes (posterior probability > 0.8); numbers in parentheses show the posterior probability. Click here for file

Additional file 9 **Ranked list of candidate driver genes for the METABRIC dataset**. rank: rank of the gene according to DriverNet, gene: gene symbol, gband: gene chromosome location and gene band, SNV.Indel: number of cases with SNV or indel in that specific gene, HLAMP: number of cases with copy number high-level amplifications, AMP: number of cases with copy number amplifications, HOMD: number of cases with copy number homozygous deletions, HETD: number of cases with copy number hemizygous deletions, covered events: the number of events (edges) connected to the gene on the left of the bipartite graph, node degree: the number of genes connected to the gene of interest in the influence graph, p.value: *P *value corrected for the multiple test using the Benjamini-Hochberg approach, CGC.status: Cancer Gene Census (CGC) membership status (1 = found in CGC, 0 = not in CGC), percentage.event: percentage of cases with genomic aberrations in the gene of interest, p.way: top pathways associated with outlying genes (posterior probability > 0.8); numbers in parentheses show the posterior probability. Click here for file

Additional file 10 **Figure showing the SNVs/indels, homozygous deletion (HOMD), and high-level amplification (HLAMP) status across the patients for the top 190 candidate driver genes (ranked from top to bottom) for the METABRIC dataset**. Genes with *P *values ≤ 0.05 are shown. Red blocks show HLAMPs and blue show HOMDs for each case. Click here for file

Additional file 11 **Ranked list of candidate driver genes for the HGS dataset**. rank: rank of the gene according to DriverNet, gene: gene symbol, gband: gene chromosome location and gene band, SNV.Indel: number of cases with SNV or indel in that specific gene, HLAMP: number of cases with copy number high-level amplifications, AMP: number of cases with copy number amplifications, HOMD: number of cases with copy number homozygous deletions, HETD: number of cases with copy number hemizygous deletions, covered events: the number of events (edges) connected to the gene on the left of the bipartite graph, node degree: the number of genes connected to the gene of interest in the influence graph, p.value: *P *value corrected for the multiple test using the Benjamini-Hochberg approach, CGC.status: Cancer Gene Census (CGC) membership status (1 = found in CGC, 0 = not in CGC), percentage.event: percentage of cases with genomic aberrations in the gene of interest, p.way: top pathways associated with outlying genes (posterior probability > 0.8); numbers in parentheses show the posterior probability. Click here for file

Additional file 12 **Figure showing the SNVs/indels, homozygous deletion (HOMD), and high-level amplification (HLAMP) status across the patients for the top 144 candidate driver genes (ranked from top to bottom) for the HGS dataset**. Genes with *P *values ≤ 0.05 are shown. Green blocks show SNVs or indels, red blocks show HLAMPs, and blue show HOMDs for each case. Click here for file

Additional file 13 **Ranked list of candidate driver genes for the TN dataset**. rank: rank of the gene according to DriverNet, gene: gene symbol, gband: gene chromosome location and gene band, SNV.Indel: number of cases with SNV or indel in that specific gene, HLAMP: number of cases with copy number high-level amplifications, AMP: number of cases with copy number amplifications, HOMD: number of cases with copy number homozygous deletions, HETD: number of cases with copy number hemizygous deletions, covered events: the number of events (edges) connected to the gene on the left of the bipartite graph, node degree: the number of genes connected to the gene of interest in the influence graph, p.value: *P *value corrected for the multiple test using the Benjamini-Hochberg approach, CGC.status: Cancer Gene Census (CGC) membership status (1 = found in CGC, 0 = not in CGC), percentage.event: percentage of cases with genomic aberrations in the gene of interest, p.way: top pathways associated with outlying genes (posterior probability > 0.8); numbers in parentheses show the posterior probability. Click here for file

Additional file 14 **Figure showing the SNVs/indels, homozygous deletion (HOMD), and high-level amplification (HLAMP) status across the patients for the top 50 candidate driver genes (ranked from top to bottom) for the TN dataset**. Genes with *P *values ≤ 0.05 are shown. Green blocks show SNVs or indels, red blocks show HLAMPs, and blue show HOMDs for each case. Click here for file

Additional file 15 **Ranked list of candidate driver genes for the GBM dataset**. rank: rank of the gene according to DriverNet, gene: gene symbol, gband: gene chromosome location and gene band, SNV.Indel: number of cases with SNV or indel in that specific gene, HLAMP: number of cases with copy number high-level amplifications, AMP: number of cases with copy number amplifications, HOMD: number of cases with copy number homozygous deletions, HETD: number of cases with copy number hemizygous deletions, covered events: the number of events (edges) connected to the gene on the left of the bipartite graph, node degree: the number of genes connected to the gene of interest in the influence graph, p.value: *P *value corrected for the multiple test using the Benjamini-Hochberg approach, CGC.status: Cancer Gene Census (CGC) membership status (1 = found in CGC, 0 = not in CGC), percentage.event: percentage of cases with genomic aberrations in the gene of interest, p.way: top pathways associated with outlying genes (posterior probability > 0.8); numbers in parentheses show the posterior probability. Click here for file

Additional file 16 **Figure showing the SNVs/indels, homozygous deletion (HOMD), and high-level amplification (HLAMP) status across the patients for the top 49 candidate driver genes (ranked from top to bottom) for the GBM dataset**. Genes with *P *values ≤0.05 are shown. Green blocks show SNVs or indels, red blocks show HLAMPs, and blue show HOMDs for each case. Click here for file

Additional file 17 **Circos plots showing outlying genes related to metabolic pathways for *PNMT *(A), *NDUFC2 *(B), and *MTAP *(C) and outlying genes related to oncogenic/tumor suppressor pathways for *ERBB2 *(D), *PAK1 *(E), and *CDKN2A *(F) genes**. Click here for file

Additional file 18 **Frequency of aberrations versus the rank of significant genes (*p *≤ 0.05) for the GBM (A), HGS (B), TN (C), and METABRIC (D) datasets**. Click here for file

Additional file 19 **Node degree in the influence graph versus the rank of significant genes (*p *≤ 0.05) for the GBM (A), HGS (B), TN (C), and METABRIC (D) datasets**. Click here for file

Additional file 20 **DriverNet performance benchmarking on GBM, TN, HGS, and METABRIC datasets when copy number amplifications (AMP) and hemizygous deletions (HETDs) were included in addition to the high-level amplifications (HLAMP) and homozygous deletions (HOMDs)**. **(A-D) **Concordance with Cancer Gene Census for DriverNet, Frequency-based, and Fisher-based approaches as a function of the top *N *ranked genes (out of 200) for the GBM, TN, HGS, and METABRIC datasets, respectively. **(E-H) **Concordance with COSMIC database (cumulative distribution of mutation prevalence in the COSMIC database) for DriverNet, Frequency-based, and Fisher-based approaches as a function of the top *N *ranked genes (out of 200) for the GBM, TN, HGS, and METABRIC datasets, respectively. Click here for file

Additional file 21 **Ranked list of candidate driver genes for the METABRIC dataset when copy number amplifications and hemizygous deletions were included in addition to the mutations, high-level amplifications, and homozygous deletions**. rank: rank of the gene according to DriverNet, gene: gene symbol, gband: gene chromosome location and gene band, SNV.Indel: number of cases with SNV or indel in that specific gene, HLAMP: number of cases with copy number high-level amplifications, AMP: number of cases with copy number amplifications, HOMD: number of cases with copy number homozygous deletions, HETD: number of cases with copy number hemizygous deletions, covered events: the number of events (edges) connected to the gene on the left of the bipartite graph, node degree: the number of genes connected to the gene of interest in the influence graph, p.value: *P *value corrected for the multiple test using the Benjamini-Hochberg approach, CGC.status: Cancer Gene Census (CGC) membership status (1 = found in CGC, 0 = not in CGC), percentage.event: percentage of cases with genomic aberrations in the gene of interest, p.way: top pathways associated with outlying genes (posterior probability > 0.8); numbers in parentheses show the posterior probability. Click here for file

Additional file 22 **Ranked list of candidate driver genes for the HGS dataset when copy number amplifications and hemizygous deletions were included in addition to the mutations, high-level amplifications, and homozygous deletions**. rank: rank of the gene according to DriverNet, gene: gene symbol, gband: gene chromosome location and gene band, SNV.Indel: number of cases with SNV or indel in that specific gene, HLAMP: number of cases with copy number high-level amplifications, AMP: number of cases with copy number amplifications, HOMD: number of cases with copy number homozygous deletions, HETD: number of cases with copy number hemizygous deletions, covered events: the number of events (edges) connected to the gene on the left of the bipartite graph, node degree: the number of genes connected to the gene of interest in the influence graph, p.value: *P *value corrected for the multiple test using the Benjamini-Hochberg approach, CGC.status: Cancer Gene Census (CGC) membership status (1 = found in CGC, 0 = not in CGC), percentage.event: percentage of cases with genomic aberrations in the gene of interest, p.way: top pathways associated with outlying genes (posterior probability > 0.8); numbers in parentheses show the posterior probability. Click here for file

Additional file 23 **Ranked list of candidate driver genes for the TN dataset when copy number amplifications and hemizygous deletions were included in addition to the mutations, high-level amplifications, and homozygous deletions**. rank: rank of the gene according to DriverNet, gene: gene symbol, gband: gene chromosome location and gene band, SNV.Indel: number of cases with SNV or indel in that specific gene, HLAMP: number of cases with copy number high-level amplifications, AMP: number of cases with copy number amplifications, HOMD: number of cases with copy number homozygous deletions, HETD: number of cases with copy number hemizygous deletions, covered events: the number of events (edges) connected to the gene on the left of the bipartite graph, node degree: the number of genes connected to the gene of interest in the influence graph, p.value: *P *value corrected for the multiple test using the Benjamini-Hochberg approach, CGC.status: Cancer Gene Census (CGC) membership status (1 = found in CGC, 0 = not in CGC), percentage.event: percentage of cases with genomic aberrations in the gene of interest, p.way: top pathways associated with outlying genes (posterior probability > 0.8); numbers in parentheses show the posterior probability. Click here for file

Additional file 24 **Ranked list of candidate driver genes for the GBM dataset when copy number amplifications and hemizygous deletions were included in addition to the mutations, high-level amplifications, and homozygous deletions**. rank: rank of the gene according to DriverNet, gene: gene symbol, gband: gene chromosome location and gene band, SNV.Indel: number of cases with SNV or indel in that specific gene, HLAMP: number of cases with copy number high-level amplifications, AMP: number of cases with copy number amplifications, HOMD: number of cases with copy number homozygous deletions, HETD: number of cases with copy number hemizygous deletions, covered events: the number of events (edges) connected to the gene on the left of the bipartite graph, node degree: the number of genes connected to the gene of interest in the influence graph, p.value: *P *value corrected for the multiple test using the Benjamini-Hochberg approach, CGC.status: Cancer Gene Census (CGC) membership status (1 = found in CGC, 0 = not in CGC), percentage.event: percentage of cases with genomic aberrations in the gene of interest, p.way: top pathways associated with outlying genes (posterior probability > 0.8); numbers in parentheses show the posterior probability. Click here for file
