# Supplementary material for: DriverNet: uncovering the impact of somatic driver mutations on transcriptional networks in cancer
Source: Genome Biol. 2012 Dec 22;13(12):R124. doi: 10.1186/gb-2012-13-12-r124 (PMC4056374; doi:10.1186/gb-2012-13-12-r124)
Supplement: Additional file 1 — Supplementary text. [file gb-2012-13-12-r124-S1.PDF]

Additional Text - Drivernet: uncovering the impact of somatic driver mutations on transcriptional networks in cancer

Ali Bashashati[1,*], Gholamreza Haffari[1,2,*], Jiarui Ding[1,3,*], Gavin Ha[1,4], Kenneth Lui[1], Jamie Rosner[1], David Huntsman[5,6], Carlos Caldas[7], Samuel Aparicio[1,5], and Sohrab P Shah[1,3,5,+]

[1]Department of Molecular Oncology, British Columbia Cancer Agency, 675 West 10th Avenue, Vancouver, BC V5Z 1L3, Canada
[2]Faculty of Information Technology, Monash University, Wellington Road, Clayton, VIC 3800, Australia
[3]Department of Computer Science, University of British Columbia, 2366 Main Mall, Vancouver, BC V6T 1Z4, Canada
[4]Bioinformatics Training Program, University of British Columbia, 570 West 7th Avenue, Vancouver, BC V5Z 4S6, Canada
[5]Department of Pathology and Laboratory Medicine, University of British Columbia, 2211 Wesbrook Mall, Vancouver, BC V6T 2B5, Canada
[6]Centre for Translational and Applied Genomics, BC Cancer Agency, 600 West 10th Avenue, Vancouver, BC V5Z 4E6 Canada
[7]Cancer Research UK, Cambridge Research Institute, Li Ka Shing Centre, Robinson Way, Cambridge, CB2 0RE, UK
[*] Equal contribution

1 Data Analysis Workflow

The driverNet algorithm needs a binary patient-mutation matrix, a binary patient-outlier matrix, and a binary matrix representing the influence graph. In this section, we describe how different matrices are prepared.

Additional File 2 shows the data processing pipeline. To obtain the patient-mutation matrix, firstly, both the tumour and normal cell DNAs are sequenced. The short reads from DNA sequencing are aligned to a human reference genome, e.g., hg18. After that, single nucleotide variants (SNVs) and small insertions and deletions (Indels) are called. Typically the called SNVs and Indels are validated by targeted high coverage sequencing. Note that we only consider the somatic mutations, which only exist in the tumour but not in the normal cells.

For somatic copy number alterations (CNA) detection, high density Affymetrix SNP6.0 GenomeWide genotyping arrays were used. Germline CNVs were predicted simultaneously and removed using HMM-Dosage [1] (`http://compbio.bccrc.ca/software/hmm-dosage/`). More details of CNA detection can also be found in Section 2 of this document text (below).

The validated somatic SNVs, indels, and called CNAs are combined to produce the patient-mutation matrix M. $M(i, j) = 1$ indicates that gene i is mutated in patient j, where mutations can take the form of SNVs, Indels and CNAs (homozygous deletions and high-level amplifications).

The gene expression in cells is measured by the mRNA level. Both microarray and RNAseq (whole transcriptome shotgun sequencing) can be used to measure the mRNA levels in cells. Microarray is cheaper and faster to measure mRNA abundance, but RNAseq provides an unbiased way to measure transcripome.

The gene expression measured from microarray or RNAseq produces the patient-expression matrix G. $G(i, j)$ represents the relative abundance of mRNA levels for gene i in patient j. For each gene, we assume the expressions across all the patients are normally distributed. Based on this assumption, the patient-expression matrix G is converted to a binary patient-outlier matrix G' where $G'(i, j) = 1$ means the expression of gene i is an outlier in patient j. The outliers for gene i are defined as those whose values are outside the two-standard deviation range of the expression values of gene i across all the patients.

The protein functional interaction network [2] is used to build the binary influence graph I. $I(i, j) = 1$ indicates that there is a path from gene i to gene j in the protein functional interaction network. Currently the influence graph has 7386 genes and 3457328 interactions.

2 Copy number analysis of Affymetrix SNP6 genotyping arrays

Copy number results for the METABRIC [1] and TNBC [3] datasets were taken directly from the respective publications. For GBM and HGS datasets, we applied the same copy number pipeline. For more details, see [3] and [1]. We obtained the raw SNP6 cel files for 120 GBM and 315 HGS tumour samples via the TCGA data portal.

2.0.1 Normalization of intensities

The Affymetrix SNP6.0 array samples were all independently normalized using the single array method CRMAv2[4]. We applied the default settings using the following tags: ACC,ra,-XY,BPN,-XY,AVG,A+B,FLN,-XY. For each sample, allelic-crosstalk calibration, probe sequence effects normalization, probe-level summarization, and PCR fragment length normalization was performed. The following annotation files were used (Feb 14, 2008): Chip definition file (GenomeWideSNP_6.Full.cdf), Unit fragment-length (GenomeWideSNP_6,Full,na24,HB20080214.ufl), Unit genome position (GenomeWideSNP_6,Full,na24,HB20080214.ugp).

2.0.2 Normalization against the reference sample

Log ratios were computed for both tumours and normals by normalizing each array independently against a reference. For each of the four tumour datasets, we generated a pooled reference from a specific normal dataset. For METABRIC and TNBC, we used 482 matched normal samples from the METABRIC study to generate a modified pooled reference (see [1] for details). For GBM and HGS, we used 270 HapMap samples [5] to generate a modified pooled reference.

2.0.3 Segmentaton and copy number analysis using HMM-Dosage

We performed copy number analysis on the four tumour datasets using HMM-Dosage [1], which is an extension of a previous method [6]. HMM-Dosage can be downloaded at `http://compbio.bccrc.ca/software/hmm-dosage/`. The algorithm was designed to detect and distinguish the complete set of somatic and germline copy number events in cancer genomes interrogated by SNP array data. The framework uses an 11-state hidden Markov model (HMM) that includes 5 levels of somatic CNAs, 5 levels of germline CNVs, and a neutral (diploid) state. The 5 somatic states are $HOMD, HETD, GAIN, AMP, HLAMP$, which represent homozygous deletion, hemizygous deletion, copy number gain, amplification and high-level amplification, respectively.

The model also requires CNV prior values which were computed as probe-level CNV frequencies. For METABRIC and TNBC, we used the 482 normal samples and an external dataset of 450 HapMap normal samples whose CNVs were predicted by [7]. For GBM and HGS, we used the 450 HapMap normal samples only.

HMM-Dosage predicts both somatic CNAs and germline CNVs simultaneously. Because we were only interested in somatic events, all CNV predictions were excluded in downstream analyses.

2.1 Gene-based copy number alterations

In order to identify the genes that are altered by copy number changes, we searched for overlap of segments with gene regions. We used gene annotations and coordinates for protein-coding genes in Ensembl 54 (hg18). For each of the four datasets, we generated a patient-by-gene copy number matrix to capture the gene alterations, $C \in \mathbb{Z}^{P \times G}$. This matrix was populated with values representing discrete copy number state calls. For each patient $p \in P$ and each gene $g \in G$, we identify segment s that overlaps g and assign $C(p, g)$ with the copy number state of s.

If gene g overlaps or is broken by a set of segments, $S = s_1, \ldots, s_k$, where $k \geq 2$, we assign $C(p, g)$ with the copy number state of the segment having max $severity$ based on the binary relation (Equation 1).

$$
\begin{aligned}
severity \;=\; & \big\{(\text{`}NEUT\text{'}, 0), \\
& (\text{`}HOMD\text{'}, 8), (\text{`}HETD\text{'}, 6), \\
& (\text{`}GAIN\text{'}, 5), (\text{`}AMP\text{'}, 6), (\text{`}HLAMP\text{'}, 8), \\
& (\text{`}CNVHOMD\text{'}, 4), (\text{`}CNVHETD\text{'}, 3), \\
& (CNVGAIN\text{'}, 2), (\text{`}CNVAMP\text{'}, 3), (\text{`}CNVHLAMP\text{'}, 4)\big\} \qquad (1)
\end{aligned}
$$

3 Datasets

Details of the datasets can be found in Table 1 of the main text. Below, we explain how the data can be accessed.

For the GBM dataset, the somatic single nucleotide variants (SNVs) and Indels list (level 3) were downloaded from TCGA data portal (`https://tcga-data.nci.nih.gov/tcga/dataAccessMatrix.htm`). The SNP6.0 CEL files (level 1) were downloaded from the data portal, and the somatic CNAs were called by HMM-Dosage (`http://compbio.bccrc.ca/software/hmm-dosage/`). The microarray gene expression data (level 3) were also download from the data portal.

The patient-mutation and outlier matrices for Metabric and TN datasets were provided in the additional material of their respective papers ([1, 3]). CNA events for both datasets were called using HMM-dosage [1].

As for the TCGA GBM dataset [8], the somatic SNVs and Indels list (level 3) were downloaded from TCGA data portal. The SNP6.0 CEL files (level 1) were downloaded from the data portal, and the somatic CNVs are called by HMM-Dosage. The microarray gene expression data (level 3) were also downloaded from the TCGA data portal.

patient-mutation and patient-outlier matrices for the above datasets can be downloaded from:
`http://compbio.bccrc.ca/software/drivernet/`

4 Fisher-based Approach [9]

Step 1: Given mutation status and expression data, unpaired Significance Analysis of Microarrays (SAM) ([10]) is used to find genes that are differentially expressed with respect to the mutation status of a particular gene across all samples. Genes with a false discovery rate (FDR) of less than 0.05 are passed to the next stage of the algorithm.

Step 2: Given expression matrix of the selected genes in the previous step, two binary matrices (one for significant overexpression and one for significant underexpression) are generated according as follows: (i) The z-score for each expression matrix element is calculated with respect to that element's row (i.e., gene specific); this is repeated for each row (gene). (ii) For the overexpressed binary matrix, any element with a z-score > 2.0 is 1 (true), otherwise the element is 0 (false); for the underexpressed binary matrix an element is 1 if the z-score < -2.0 and 0 otherwise. Then, Fisher's exact P value is calculated for each gene in the expression matrix by populating a 2 x 2 contingency table with a binary expression vector (category 1) and the mutation vector (category 2); this process is repeated for each binary expression vector from the binary expression matrix. This calculation allow us to recover only genes that had drastic mutation-correlated over- and underexpression and to assign each correlation with an exact P value. Mutation-correlated over- and underexpressed genes with a value of $P < 0.01$ (Fisher's exact test) and an FDR of less than 0.05 are considered as candidate drivers and were ranked according to the total number of outlying genes including both the overexpressed and underexpressed genes. The entire process is repeated once for each mutated gene [9].

5 Network and Pathway Analysis

For network and pathway analysis, we used model-based gene set analysis (MGSA) R Bioconductor package. MGSA employs probabilistic inference via a Metropolis-Hasting algorithm to estimate the probability of categories to be active. The MGSA approach naturally takes category overlap into account and avoids the need for multiple testing corrections met in single-category enrichment analysis. More details of the procedure can be found in the original publication [11]. The gene sets for the MGSA analysis were built based on the protein functional interaction network

taken from [2]. Pathways for which the posterior probability of enrichment is more than 0.8 were reported in different sections of the manuscript and in the Additional files. Significant pathways ($posterior\ probablity >= 0.8$) were exported and analyzed using EnrichmentMap [12] to determine relationships between pathways in Figure 5. For this analysis, gene sets in GMT format were provided by the Reactome FI authors (Wu, personal communication). For visualization of the enrichment maps EnrichmentMap Cytoscape plugin [12] in Cytoscape v2.8.1 [13].

Description of additional files

Additional file 1: Supplementary text

Additional file 2: Data analysis workflow

Additional file 3: Ranked list of candidate driver genes by YounSimon approach for the GBM2 dataset; rank: rank of the gene, gene: gene symbol, p.value: p-value, p.adjust: adjusted p-value using the Benjamini-Hochberg approach.

Additional file 4: Ranked list of candidate driver genes for the GBM2 dataset; rank: rank of the gene according to drivernet, gene: gene symbol, gband: gene chromosome location and gene band, SNV.Indel: number of cases with SNV or indel in that specific gene, HLAMP: number of cases with copy number high level amplifications, AMP: number of cases with copy number amplifications, HOMD: number of cases with copy number homozygous deletions, HETD: number of cases with copy number hemizygous deletions, covered_events: the number of events (edges) connected to the gene on the left hand side of the bipartite graph, node degree: the number of genes connected to the gene of interest in the influence graph, p.value: p-value corrected for multiple test using the Benjamini-Hochberg approach, CGC.status: cencer gene census (CGC) membership status (1=found in CGC, 0=not in CGC), percentage.event: percentage of cases with genomic aberrations in the gene of interest, p.way: top pathways associated with outlying genes (posterior probability>0.8); numbers in parentheses show the posterior probability.

Additional file 5: Ranked list of candidate driver genes by YounSimon approach for the TN2 dataset; rank: rank of the gene, gene: gene symbol, p.value: p-value, p.adjust: adjusted p-value using the Benjamini-Hochberg approach.

Additional file 6: Ranked list of candidate driver genes for the TN2 dataset; rank: rank of the gene according to drivernet, gene: gene symbol, gband: gene chromosome location and gene band, SNV.Indel: number of cases with SNV or indel in that specific gene, HLAMP: number of cases with copy number high level amplifications, AMP: number of cases with copy number amplifications, HOMD: number of cases with copy number homozygous deletions, HETD: number of cases with copy number hemizygous deletions, covered_events: the number of events (edges) connected to the gene on the left hand side of the bipartite graph, node degree: the number of genes connected to the gene of interest in the influence graph, p.value: p-value corrected for multiple test using the Benjamini-Hochberg approach, CGC.status: cencer gene census (CGC) membership status (1=found in CGC, 0=not in CGC), percentage.event: percentage of cases with genomic aberrations in the gene of interest, p.way: top pathways associated with outlying genes (posterior probability>0.8); numbers in parentheses show the posterior probability.

Additional file 7: Ranked list of candidate driver genes by YounSimon approach for the HGS2 dataset; rank: rank of the gene, gene: gene symbol, p.value: p-value, p.adjust: adjusted p-value using the Benjamini-Hochberg approach.

Additional file 8: Ranked list of candidate driver genes for the HGS2 dataset; rank: rank of the gene according to drivernet, gene: gene symbol, gband: gene chromosome location and gene band, SNV.Indel: number of cases with SNV or indel in that specific gene, HLAMP: number of cases with copy number high level amplifications, AMP: number of cases with copy number amplifications, HOMD: number of cases with copy number homozygous deletions, HETD: number of cases with copy number hemizygous deletions, covered_events: the number of events (edges) connected to the gene on the left hand side of the bipartite graph, node degree: the number of genes connected to the gene of interest in the influence graph, p.value: p-value corrected for multiple test using the Benjamini-Hochberg approach, CGC.status: cencer gene census (CGC) membership status (1=found in CGC, 0=not in CGC), percentage.event: percentage of cases with genomic aberrations in the gene of interest, p.way: top pathways associated with outlying genes (posterior probability>0.8); numbers in parentheses show the posterior probability.

Additional file 9: Ranked list of candidate driver genes for the Metabric dataset; rank: rank of the gene according to drivernet, gene: gene symbol, gband: gene chromosome location and gene band, SNV.Indel: number of cases with SNV or indel in that specific gene, HLAMP: number of cases with copy number high level amplifications, AMP: number of cases with copy number amplifications, HOMD: number of cases with copy number homozygous deletions, HETD: number of cases with copy number hemizygous deletions, covered_events: the number of events (edges) connected to the gene on the left hand side of the bipartite graph, node degree: the number of genes connected to the gene of interest in the influence graph, p.value: p-value corrected for multiple test using the Benjamini-Hochberg approach, CGC.status: cencer gene census (CGC) membership status (1=found in CGC, 0=not in CGC), percentage.event: percentage of cases with genomic aberrations in the gene of interest, p.way: top pathways associated with outlying genes (posterior probability>0.8); numbers in parentheses show the posterior probability.

Additional file 10: Figure showing the SNVs/indels, homozygous deletion (HOMD), and high level amplification (HLAMP) status across the patients for the top 190 candidate driver genes (ranked from top to bottom) for the METABRIC dataset. Genes with p-values $<=$ 0.05 are shown. Red blocks show HLAMPs, and blue show HOMDs for each case.

Additional file 11: Ranked list of candidate driver genes for the HGS dataset; rank: rank of the gene according to drivernet, gene: gene symbol, gband: gene chromosome location and gene band, SNV.Indel: number of cases with SNV or indel in that specific gene, HLAMP: number of cases with copy number high level amplifications, AMP: number of cases with copy number amplifications, HOMD: number of cases with copy number homozygous deletions, HETD: number of cases with copy number hemizygous deletions, covered_events: the number of events (edges) connected to the gene on the left hand side of the bipartite graph, node degree: the number of genes connected to the gene of interest in the influence graph, p.value: p-value corrected for multiple test using the Benjamini-Hochberg approach, CGC.status: cencer gene census (CGC) membership status (1=found in CGC, 0=not in CGC), percentage.event: percentage of cases with genomic aberrations in the gene of interest, p.way: top pathways associated with outlying genes (posterior probability$>$0.8); numbers in parentheses show the posterior probability.

Additional file 12: Figure showing the SNVs/indels, homozygous deletion (HOMD), and high level amplification (HLAMP) status across the patients for the top 144 candidate driver genes (ranked from top to bottom) for the HGS dataset. Genes with p-values $<=$ 0.05 are shown. Green blocks show SNVs or indels, red blocks show HLAMPs, and blue show HOMDs for each case.

Additional file 13: Ranked list of candidate driver genes for the TN dataset; rank: rank of the gene according to drivernet, gene: gene symbol, gband: gene chromosome location and gene band, SNV.Indel: number of cases with SNV or indel in that specific gene, HLAMP: number of cases with copy number high level amplifications, AMP: number of cases with copy number amplifications, HOMD: number of cases with copy number homozygous deletions, HETD: number of cases with copy number hemizygous deletions, covered_events: the number of events (edges) connected to the gene on the left hand side of the bipartite graph, node degree: the number of genes connected to the gene of interest in the influence graph, p.value: p-value corrected for multiple test using the Benjamini-Hochberg approach, CGC.status: cencer gene census (CGC) membership status (1=found in CGC, 0=not in CGC), percentage.event: percentage of cases with genomic aberrations in the gene of interest, p.way: top pathways associated with outlying genes (posterior probability$>$0.8); numbers in parentheses show the posterior probability.

Additional file 14: Figure showing the SNVs/indels, homozygous deletion (HOMD), and high level amplification (HLAMP) status across the patients for the top 50 candidate driver genes (ranked from top to bottom) for the TN dataset. Genes with p-values $<=$ 0.05 are shown. Green blocks show SNVs or indels, red blocks show HLAMPs, and blue show HOMDs for each case.

Additional file 15: Ranked list of candidate driver genes for the GBM dataset; rank: rank of the gene according to drivernet, gene: gene symbol, gband: gene chromosome location and gene band, SNV.Indel: number of cases with SNV or indel in that specific gene, HLAMP: number of cases with copy number high level amplifications, AMP: number of cases with copy number amplifications, HOMD: number of cases with copy number homozygous deletions, HETD: number of cases with copy number hemizygous deletions, covered_events: the number of events (edges) connected to the gene on the left hand side of the bipartite graph, node degree: the number of genes connected to the gene of interest in the influence graph, p.value: p-value corrected for multiple test using the Benjamini-Hochberg approach, CGC.status: cencer gene census (CGC) membership status (1=found in CGC, 0=not in CGC), percentage.event: percentage of cases with genomic aberrations in the gene of interest, p.way: top pathways associated with outlying genes (posterior probability$>$0.8); numbers in parentheses show the posterior probability.

Additional file 16: Figure showing the SNVs/indels, homozygous deletion (HOMD), and high level amplification (HLAMP) status across the patients for the top 49 candidate driver genes (ranked from top to bottom) for the GBM dataset. Genes with p-values $<= 0.05$ are shown. Green blocks show SNVs or indels, red blocks show HLAMPs, and blue show HOMDs for each case.

Additional file 17: Circos plots showing outlying genes related to metabolic pathways for PNMT (A), NDUFC2 (B), and MTAP (C) and outlying genes related to oncogenic/tumour suppressor pathways for ERBB2 (D), PAK1 (E), and CDKN2A (F) genes.

Additional file 18: Frequency of aberrations versus the rank of significant genes ($p <= 0.05$) for the GBM (A), HGS (B), TN (C), and Metabric (D) datasets

Additional file 19: Node degree in the influence graph versus the rank of significant genes ($p <= 0.05$) for the GBM (A), HGS (B), TN (C), and Metabric (D) datasets

Additional file 20: DriverNet performance benchmarking on GBM, TN, HGS, and METABRIC datasets when copy number amplifications (AMP) and hemizygous deletions (HETD) were included in addition to the high level amplifications (HLAMP) and homozygous deletions (HOMD). (A-D) Concordance with Cancer Gene Census for driverNet, Frequency-based and Fisher-based approaches as a function of top 'N' ranked genes (out of 200) for GBM, TN HGS, and METABRIC datasets, respectively. (E-H) Concordance with COSMIC database (cumulative distribution of mutation prevalence in the COSMIC database) for driverNet, Frequency-based and Fisher-based approaches as a function of top 'N' ranked genes (out of 200) for GBM, TN, HGS, and METABRIC datasets, respectively.

Additional file 21: Ranked list of candidate driver genes for the METABRIC dataset when copy number amplifications and hemizygous deletions were included in addition to the mutations and high level amplifications and homozygous deletions; rank: rank of the gene according to drivernet, gene: gene symbol, gband: gene chromosome location and gene band, SNV.Indel: number of cases with SNV or indel in that specific gene, HLAMP: number of cases with copy number high level amplifications, AMP: number of cases with copy number amplifications, HOMD: number of cases with copy number homozygous deletions, HETD: number of cases with copy number hemizygous deletions, covered_events: the number of events (edges) connected to the gene on the left hand side of the bipartite graph, node degree: the number of genes connected to the gene of interest in the influence graph, p.value: p-value corrected for multiple test using the Benjamini-Hochberg approach, CGC.status: cencer gene census (CGC) membership status (1=found in CGC, 0=not in CGC), percentage.event: percentage of cases with genomic aberrations in the gene of interest, p.way: top pathways associated with outlying genes (posterior probability>0.8); numbers in parentheses show the posterior probability.

Additional file 22: Ranked list of candidate driver genes for the HGS dataset when copy number amplifications and hemizygous deletions were included in addition to the mutations and high level amplifications and homozygous deletions; rank: rank of the gene according to drivernet, gene: gene symbol, gband: gene chromosome location and gene band, SNV.Indel: number of cases with SNV or indel in that specific gene, HLAMP: number of cases with copy number high level amplifications, AMP: number of cases with copy number amplifications, HOMD: number of cases with copy number homozygous deletions, HETD: number of cases with copy number hemizygous deletions, covered_events: the number of events (edges) connected to the gene on the left hand side of the bipartite graph, node degree: the number of genes connected to the gene of interest in the influence graph, p.value: p-value corrected for multiple test using the Benjamini-Hochberg approach, CGC.status: cencer gene census (CGC) membership status (1=found in CGC, 0=not in CGC), percentage.event: percentage of cases with genomic aberrations in the gene of interest, p.way: top pathways associated with outlying genes (posterior probability>0.8); numbers in parentheses show the posterior probability.

Additional file 23: Ranked list of candidate driver genes for the TN dataset when copy number amplifications and hemizygous deletions were included in addition to the mutations and high level amplifications and homozygous deletions; rank: rank of the gene according to drivernet, gene: gene symbol, gband: gene chromosome location and gene band, SNV.Indel: number of cases with SNV or indel in that specific gene, HLAMP: number of cases with copy number high level amplifications, AMP: number of cases with copy number amplifications, HOMD: number of cases with copy number homozygous deletions, HETD: number of cases with copy number hemizygous deletions, covered_events: the number of events (edges) connected to the gene on the left hand side of the bipartite graph, node degree: the number of genes connected to the gene of interest in the influence graph, p.value: p-value corrected for multiple test using the Benjamini-Hochberg approach, CGC.status: cencer gene census (CGC) membership status (1=found in CGC, 0=not in CGC), percentage.event: percentage of cases with genomic aberrations in the gene of interest, p.way: top pathways associated with outlying genes (posterior probability>0.8); numbers in parentheses show the posterior probability.

Additional file 24: Ranked list of candidate driver genes for the GBM dataset when copy number amplifications and hemizygous deletions were included in addition to the mutations and high level amplifications and homozygous deletions; rank: rank of the gene according to drivernet, gene: gene symbol, gband: gene chromosome location and gene band, SNV.Indel: number of cases with SNV or indel in that specific gene, HLAMP: number of cases with copy number high level amplifications, AMP: number of cases with copy number amplifications, HOMD: number of cases with copy number homozygous deletions, HETD: number of cases with copy number hemizygous deletions, covered_events: the number of events (edges) connected to the gene on the left hand side of the bipartite graph, node degree: the number of genes connected to the gene of interest in the influence graph, p.value: p-value corrected for multiple test using the Benjamini-Hochberg approach, CGC.status: cencer gene census (CGC) membership status (1=found in CGC, 0=not in CGC), percentage.event: percentage of cases with genomic aberrations in the gene of interest, p.way: top pathways associated with outlying genes (posterior probability>0.8); numbers in parentheses show the posterior probability.

References

[1] Curtis C, Shah SP, Chin SF, Turashvili G, Rueda OM, Dunning MJ, Speed D, Lynch AG, Samarajiwa S, Yuan Y, Graf S, Ha G, Haffari G, Bashashati A, Russell R, McKinney S, the METABRIC GROUP, Langerod A, Green A, Provenzano E, Wishart G, Pinder S, Watson P, Markowetz F, Murphy L, Ellis I, Purushotham A, Borresen-Dale AL, Brenton JD, Tavare S, , et al.: **The genomic and transcriptomic architecture of 2,000 breast tumours reveals novel subgroups**. *Nature* 2012, **486**:346–352.

[2] Wu G, Feng X, Stein L: **A human functional protein interaction network and its application to cancer data analysis.** *Genome Biology* 2010, **11**:R53.

[3] Shah S, Roth A, Goya R, Oloumi A, Ha G, Zhao Y, Turashvili G, Ding J, Tse K, Haffari G, Bashashati A, Prentice L, Khattra J, Burleigh A, Yap D, Bernard V, McPherson A, Shumansky K, Crisan A, Giuliany R, Heravi-Moussavi A, Rosner J, Lai D, Birol I, Varhol R, Tam A, Dhalla N, Zeng T, Ma K, Chan S, et al.: **The clonal and mutational evolution spectrum of primary triple-negative breast cancers**. *Nature* 2012, **486**:395–399.

[4] Bengtsson H, Wirapati P, Speed TP: **A single-array preprocessing method for estimating full-resolution raw copy numbers from all Affymetrix genotyping arrays including GenomeWideSNP 5 & 6**. *Bioinformatics* 2009, **25**(17):2149–56.

[5] International HapMap Consortium: **A second generation human haplotype map of over 3.1 million SNPs**. *Nature* 2007, **449**(7164):851–61.

[6] Shah SP, Xuan X, DeLeeuw RJ, Khojasteh M, Lam WL, Ng R, Murphy KP: **Integrating copy number polymorphisms into array CGH analysis using a robust HMM**. *Bioinformatics* 2006, **22**(14):e431–9.

[7] Conrad DF, Pinto D, Redon R, Feuk L, Gokcumen O, Zhang Y, Aerts J, Andrews TD, Barnes C, Campbell P, Fitzgerald T, Hu M, Ihm CH, Kristiansson K, Macarthur DG, Macdonald JR, Onyiah I, Pang AWC, Robson S, Stirrups K, Valsesia A, Walter K, Wei J, Wellcome Trust Case Control Consortium, Tyler-Smith C, Carter NP, Lee C, Scherer SW, Hurles ME: **Origins and functional impact of copy number variation in the human genome**. *Nature* 2010, **464**(7289):704–12.

[8] Cancer Genome Atlas Research Network: **Integrated genomic analyses of ovarian carcinoma**. *Nature* 2011, **474**:609–615.

[9] Masica D, Karchin R: **Correlation of Somatic Mutation and Expression Identifies Genes Important in Human Glioblastoma Progression and Survival**. *Cancer Research* 2011, **71**:4550.

[10] Tusher V, Tibshirani R, Chu G: **Significance analysis of microarrays applied to the ionizing radiation response**. *Proceedings of the National Academy of Sciences* 2001, **98**(9):5116.

[11] Bauer S, Gagneur J, Robinson P: **GOing Bayesian: model-based gene set analysis of genome-scale data**. *Nucleic acids research* 2010, **38**(11):3523–3532.

[12] Merico D, Isserlin R, Stueker O, Emili A, Bader G: **Enrichment map: a network-based method for gene-set enrichment visualization and interpretation**. *PLoS One* 2010, **5**:e13984.

[13] Smoot ME, Ono K, Ruscheinski J, Wang PL, Ideker T: **Cytoscape 2.8: new features for data integration and network visualization**. *Bioinformatics* 2011, **27**(3):431–432.
