# Supplementary figures and images for: DriverNet: uncovering the impact of somatic driver mutations on transcriptional networks in cancer
Source: Genome Biol. 2012 Dec 22;13(12):R124. doi: 10.1186/gb-2012-13-12-r124 (PMC4056374; doi:10.1186/gb-2012-13-12-r124)

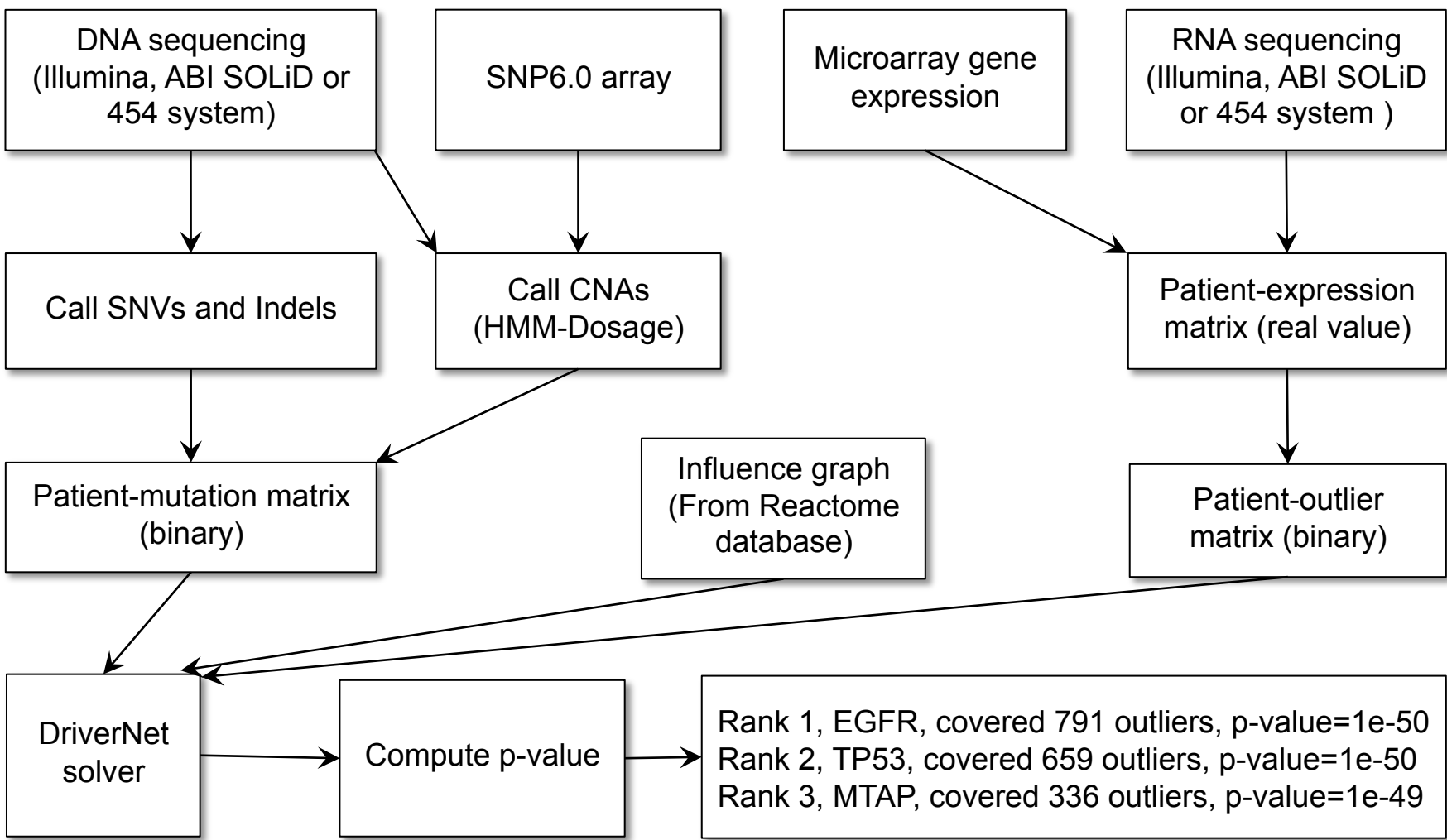

Supplement: Additional file 2 — Data analysis workflow. [file gb-2012-13-12-r124-S2.PDF]

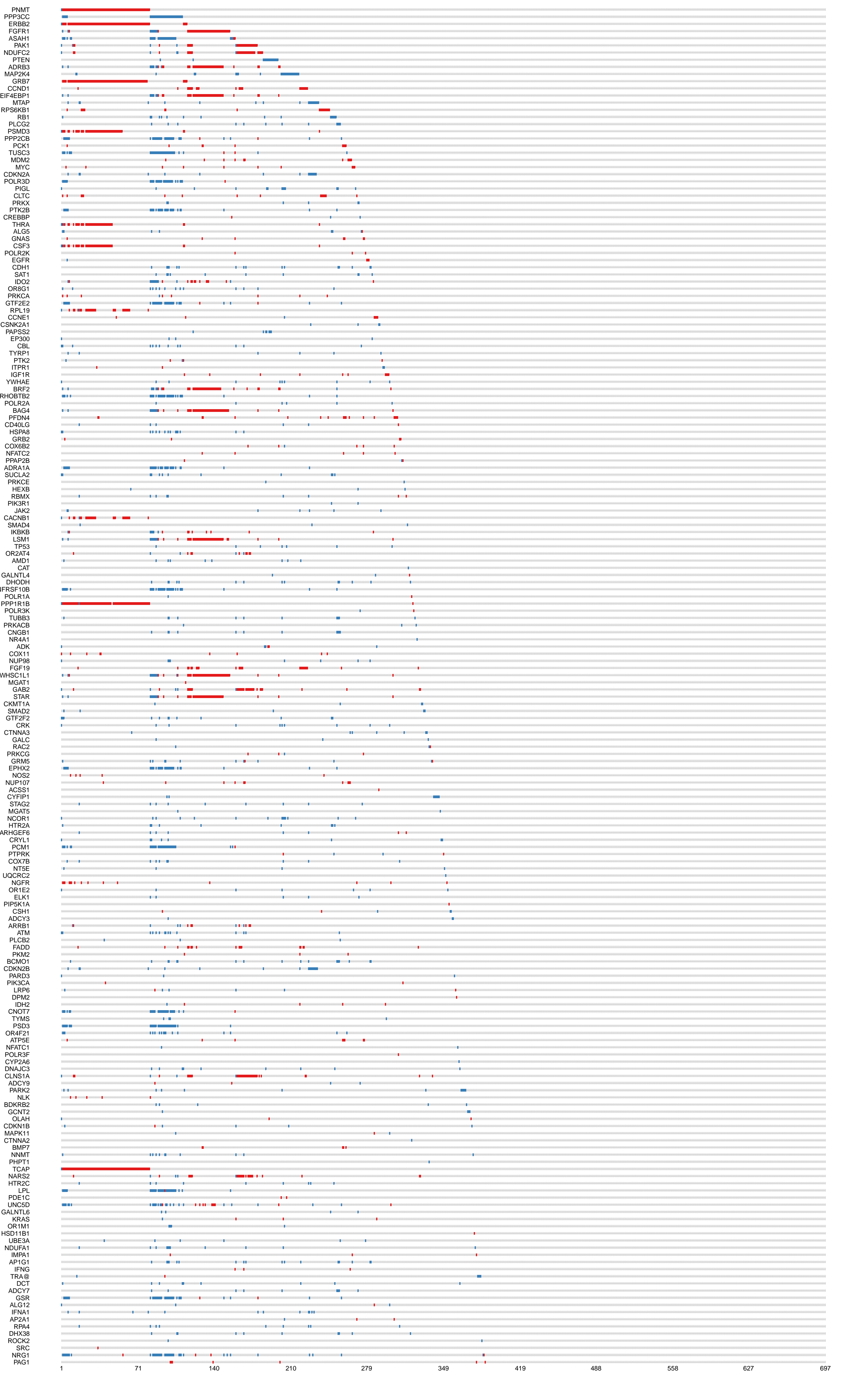

Supplement: Additional file 10 — Figure showing the SNVs/indels, homozygous deletion (HOMD), and high-level amplification (HLAMP) status across the patients for the top 190 candidate driver genes (ranked from top to bottom) for the METABRIC dataset. Genes with P values ≤ 0.05 are shown. Red blocks show HLAMPs and blue show HOMDs for each case. [file gb-2012-13-12-r124-S10.PDF]

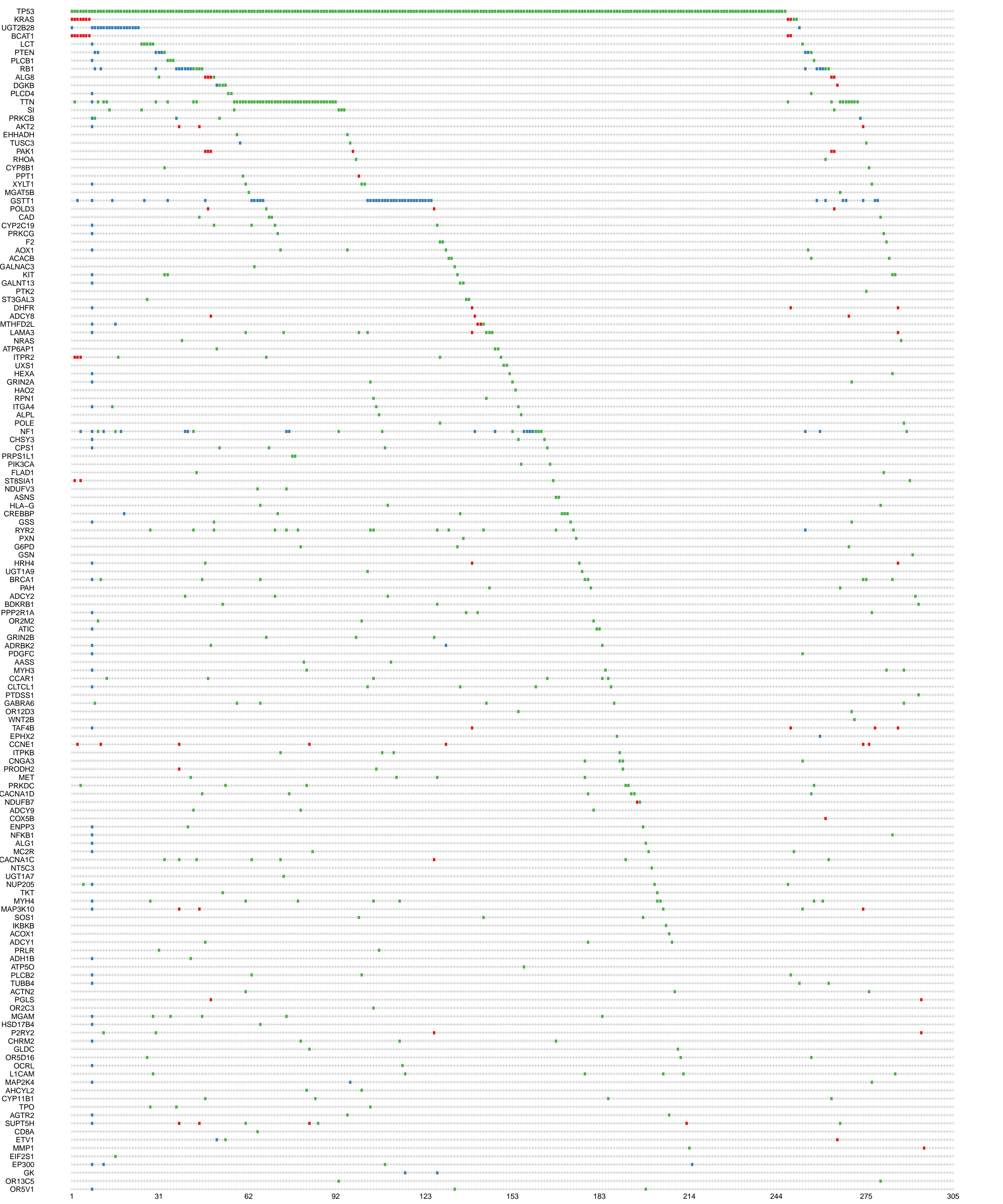

Supplement: Additional file 12 — Figure showing the SNVs/indels, homozygous deletion (HOMD), and high-level amplification (HLAMP) status across the patients for the top 144 candidate driver genes (ranked from top to bottom) for the HGS dataset. Genes with P values ≤ 0.05 are shown. Green blocks show SNVs or indels, red blocks show HLAMPs, and blue show HOMDs for each case. [file gb-2012-13-12-r124-S12.PDF]

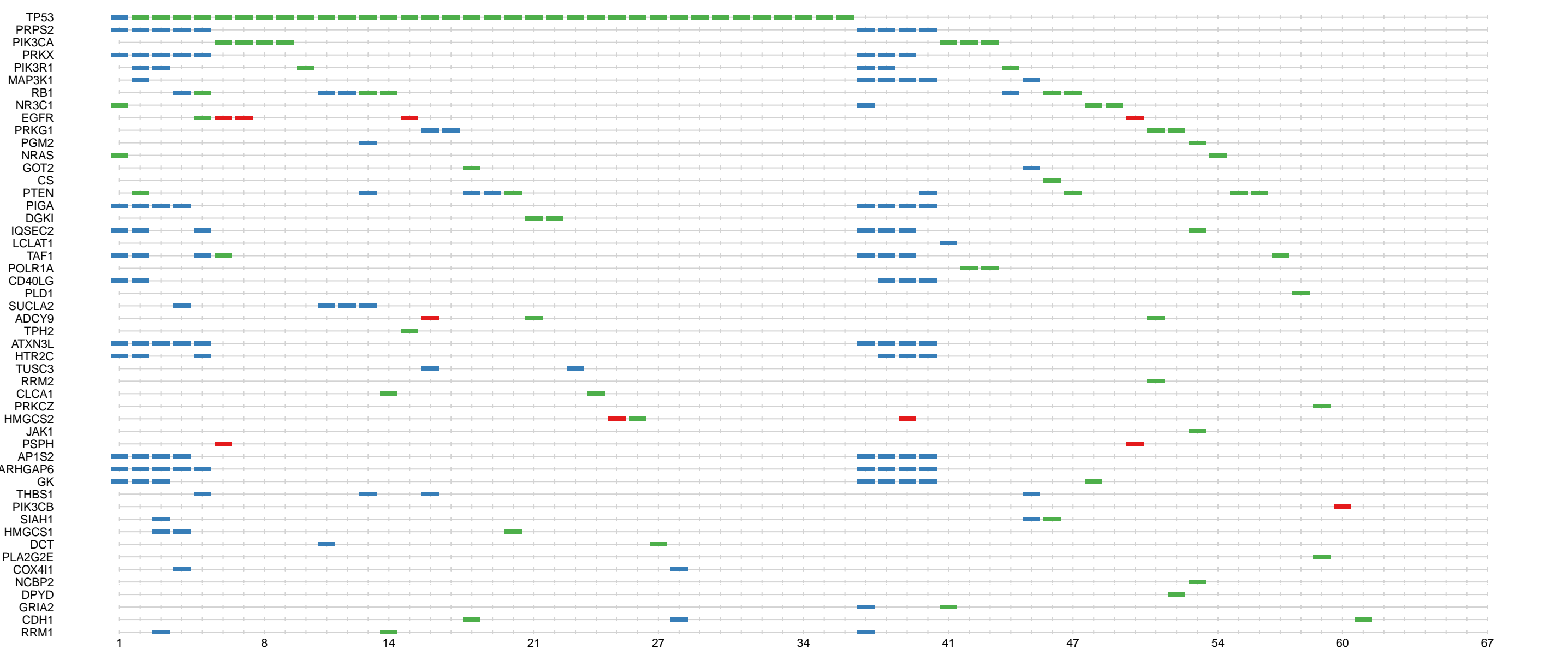

Supplement: Additional file 14 — Figure showing the SNVs/indels, homozygous deletion (HOMD), and high-level amplification (HLAMP) status across the patients for the top 50 candidate driver genes (ranked from top to bottom) for the TN dataset. Genes with P values ≤ 0.05 are shown. Green blocks show SNVs or indels, red blocks show HLAMPs, and blue show HOMDs for each case. [file gb-2012-13-12-r124-S14.PDF]

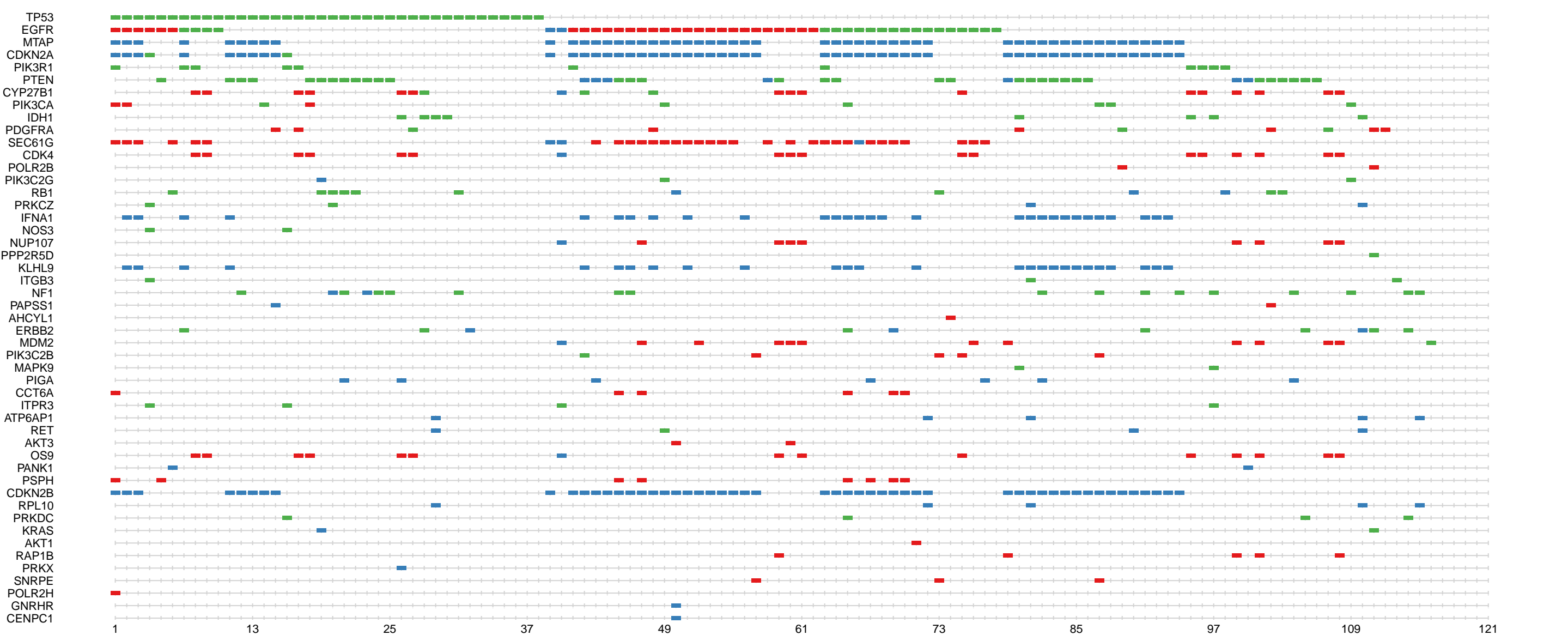

Supplement: Additional file 16 — Figure showing the SNVs/indels, homozygous deletion (HOMD), and high-level amplification (HLAMP) status across the patients for the top 49 candidate driver genes (ranked from top to bottom) for the GBM dataset. Genes with P values ≤0.05 are shown. Green blocks show SNVs or indels, red blocks show HLAMPs, and blue show HOMDs for each case. [file gb-2012-13-12-r124-S16.PDF]

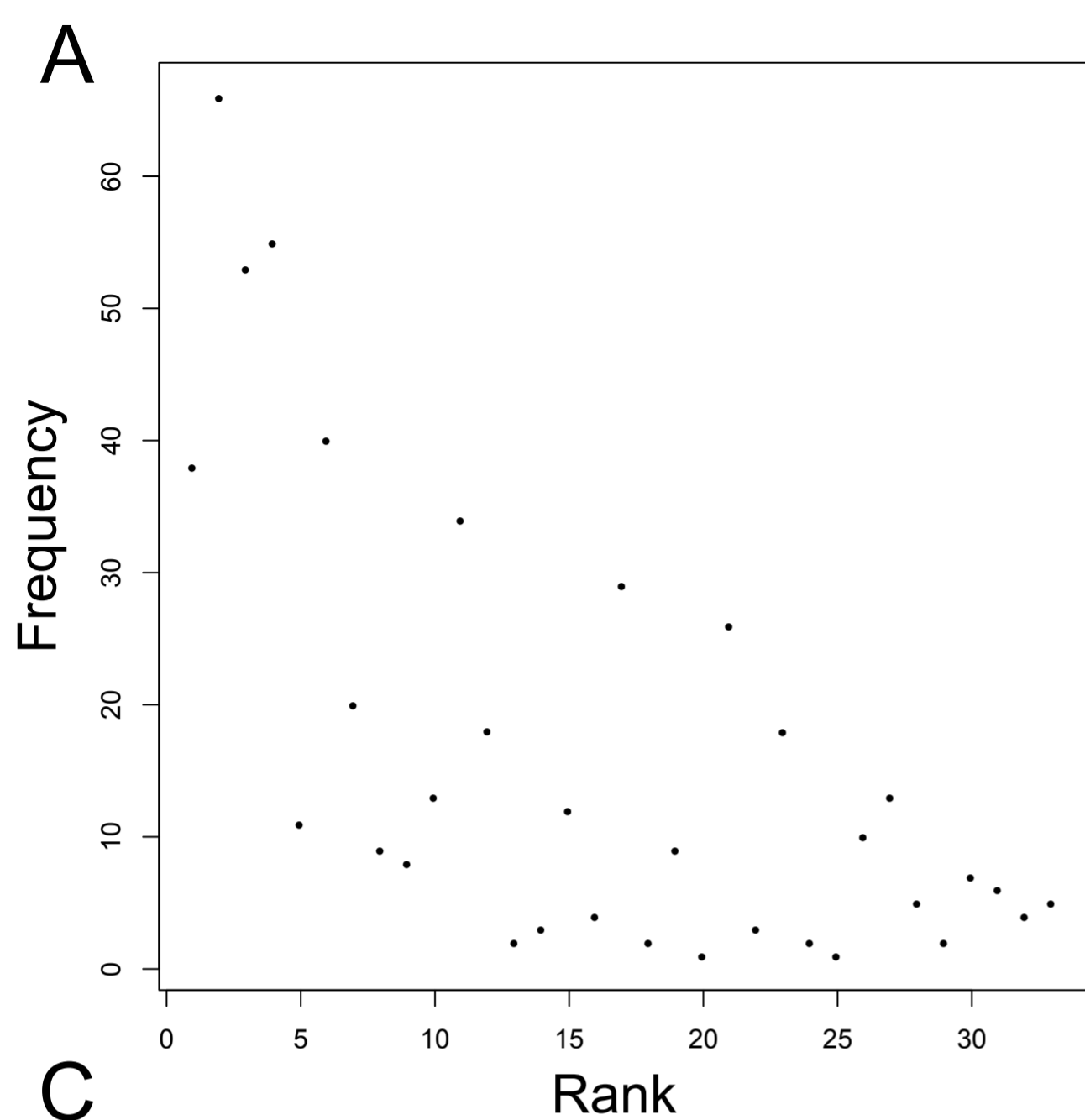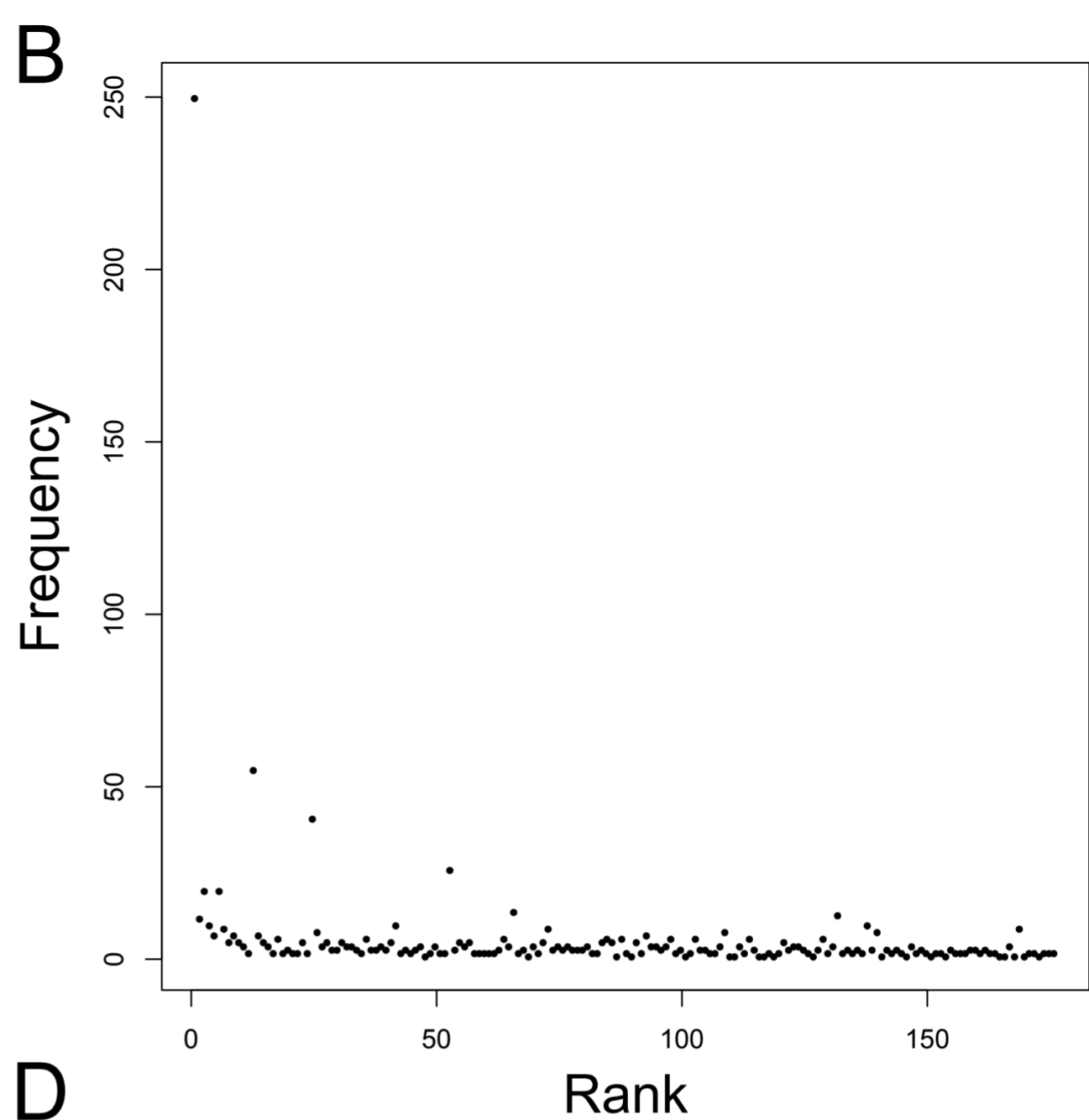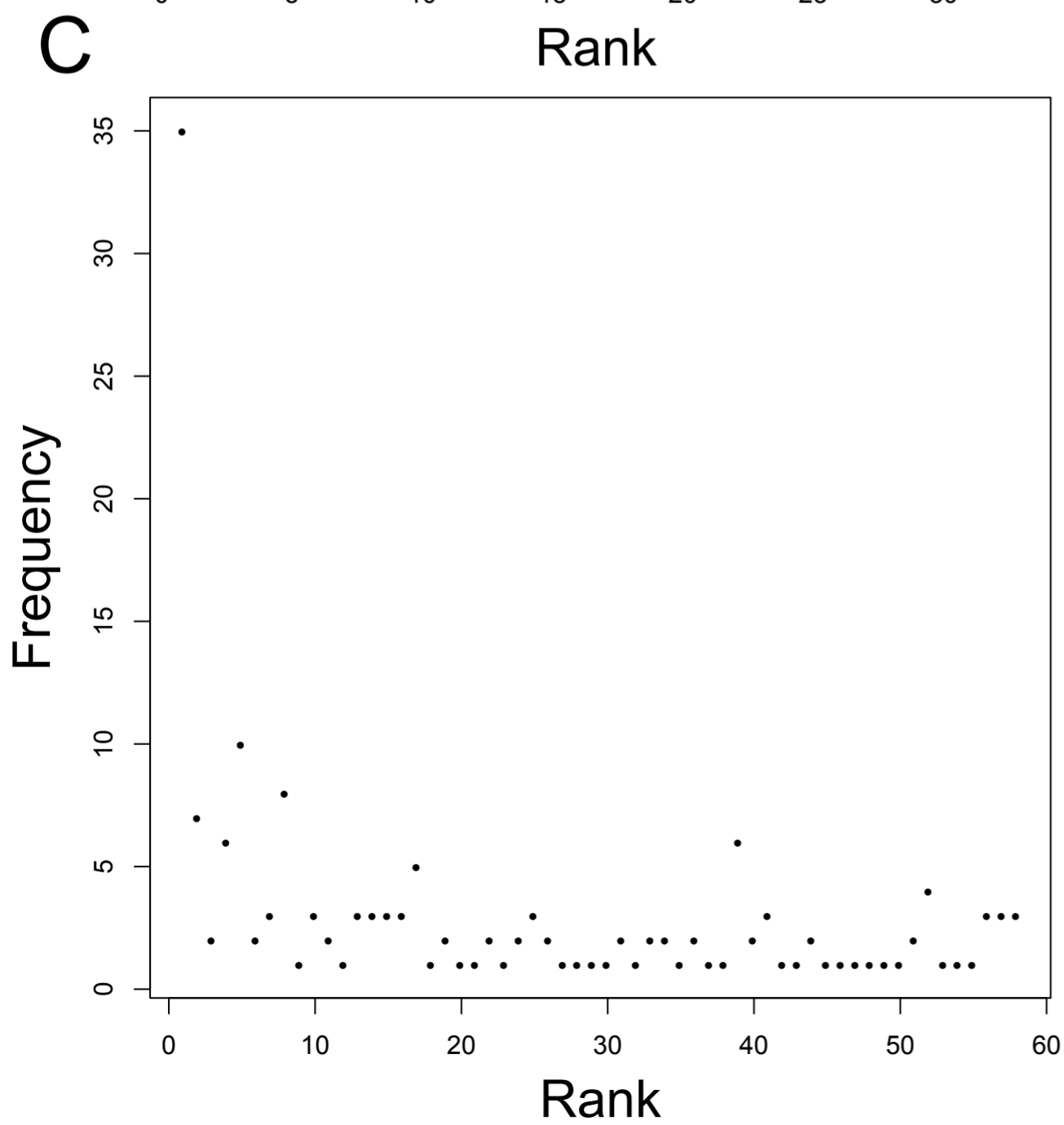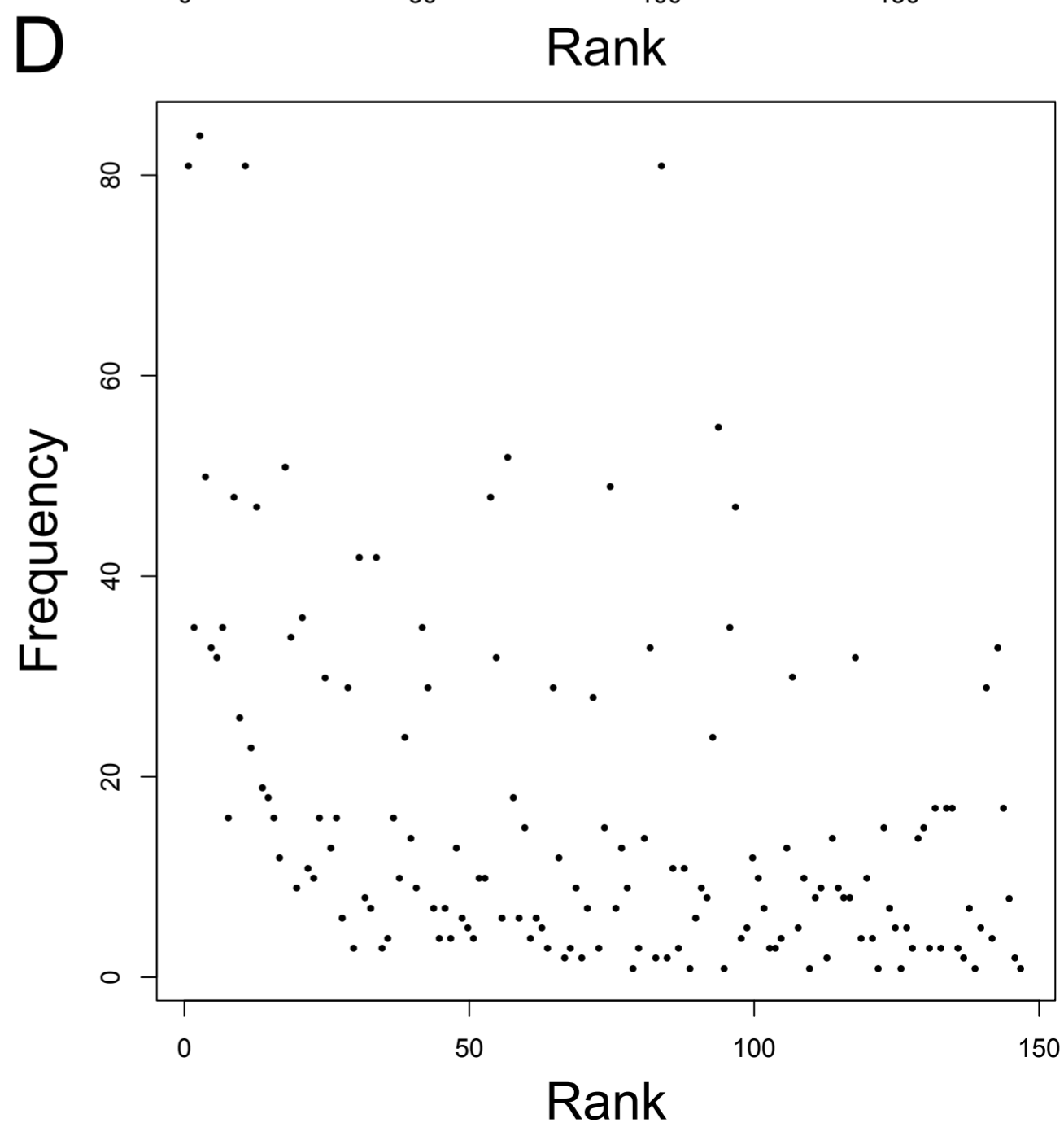

Supplement: Additional file 18 — Frequency of aberrations versus the rank of significant genes (p ≤ 0.05) for the GBM (A), HGS (B), TN (C), and METABRIC (D) datasets. [file gb-2012-13-12-r124-S18.PDF]

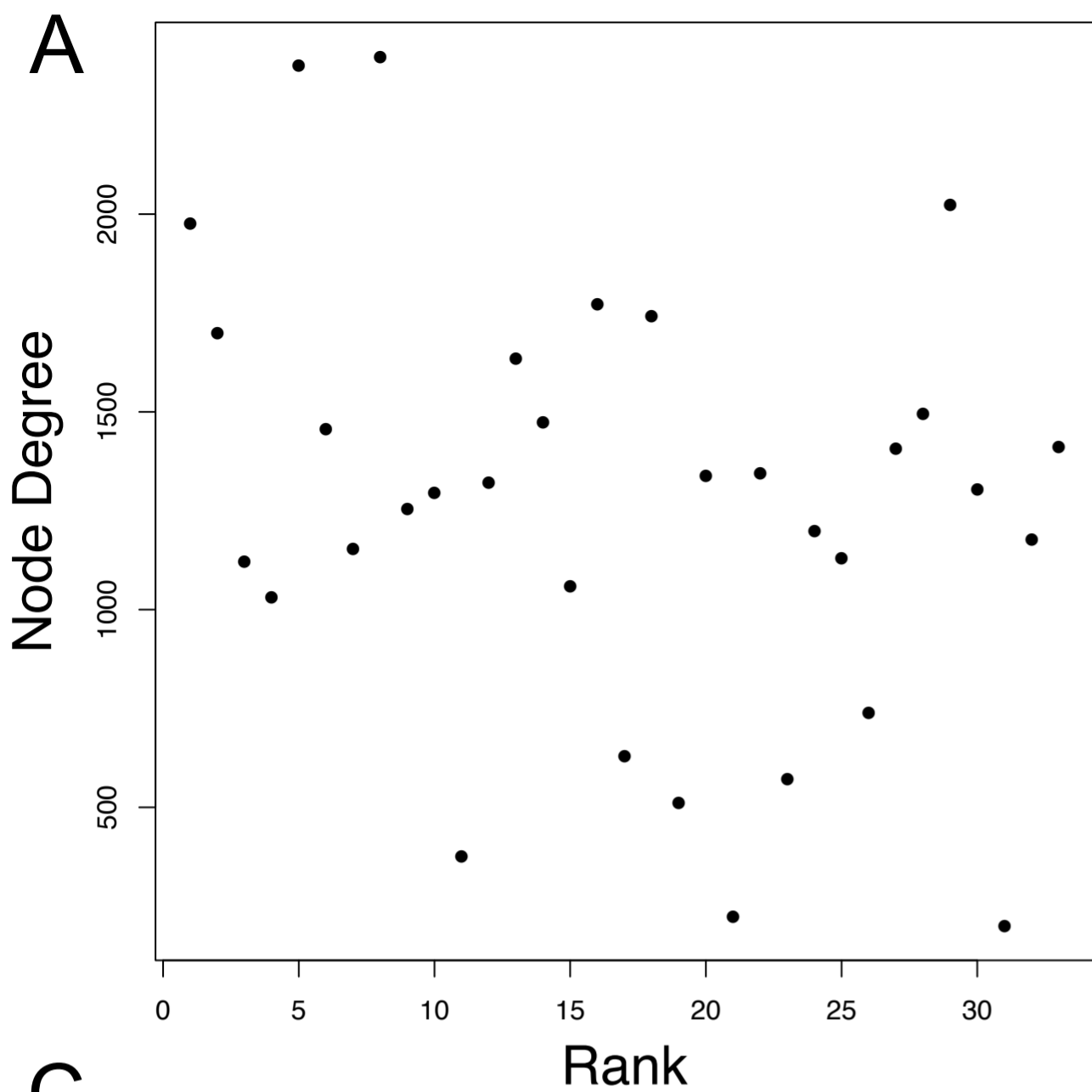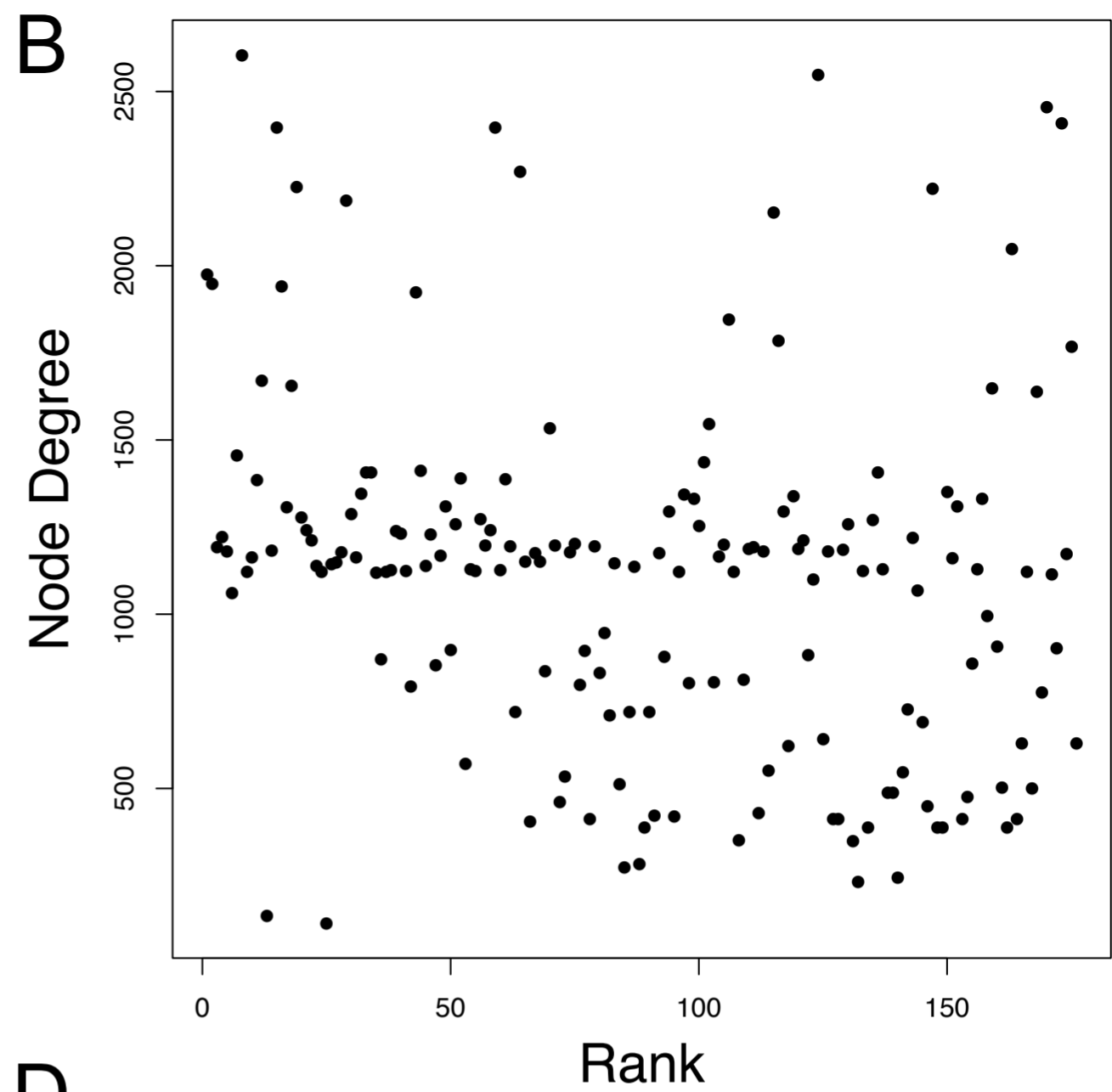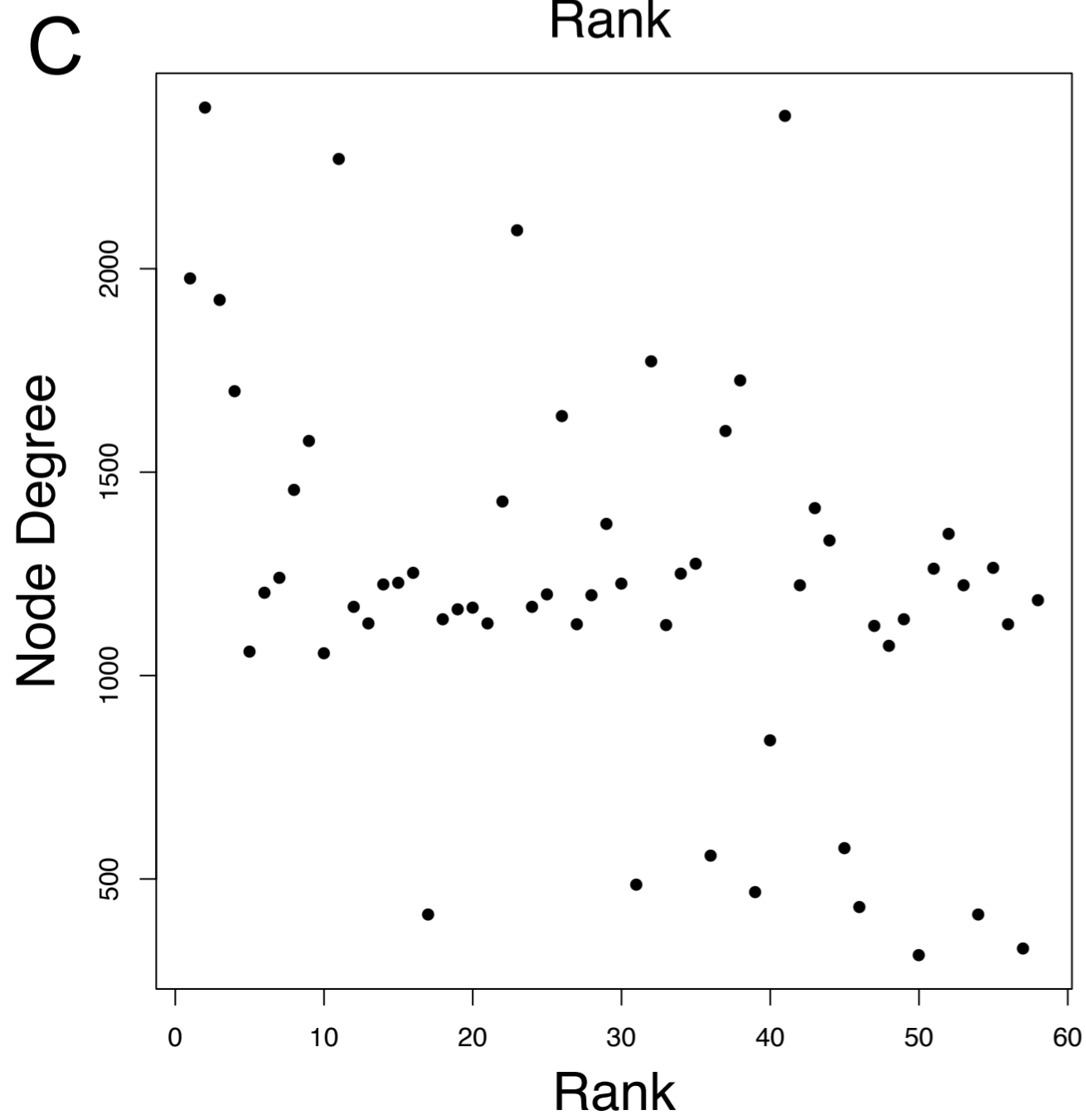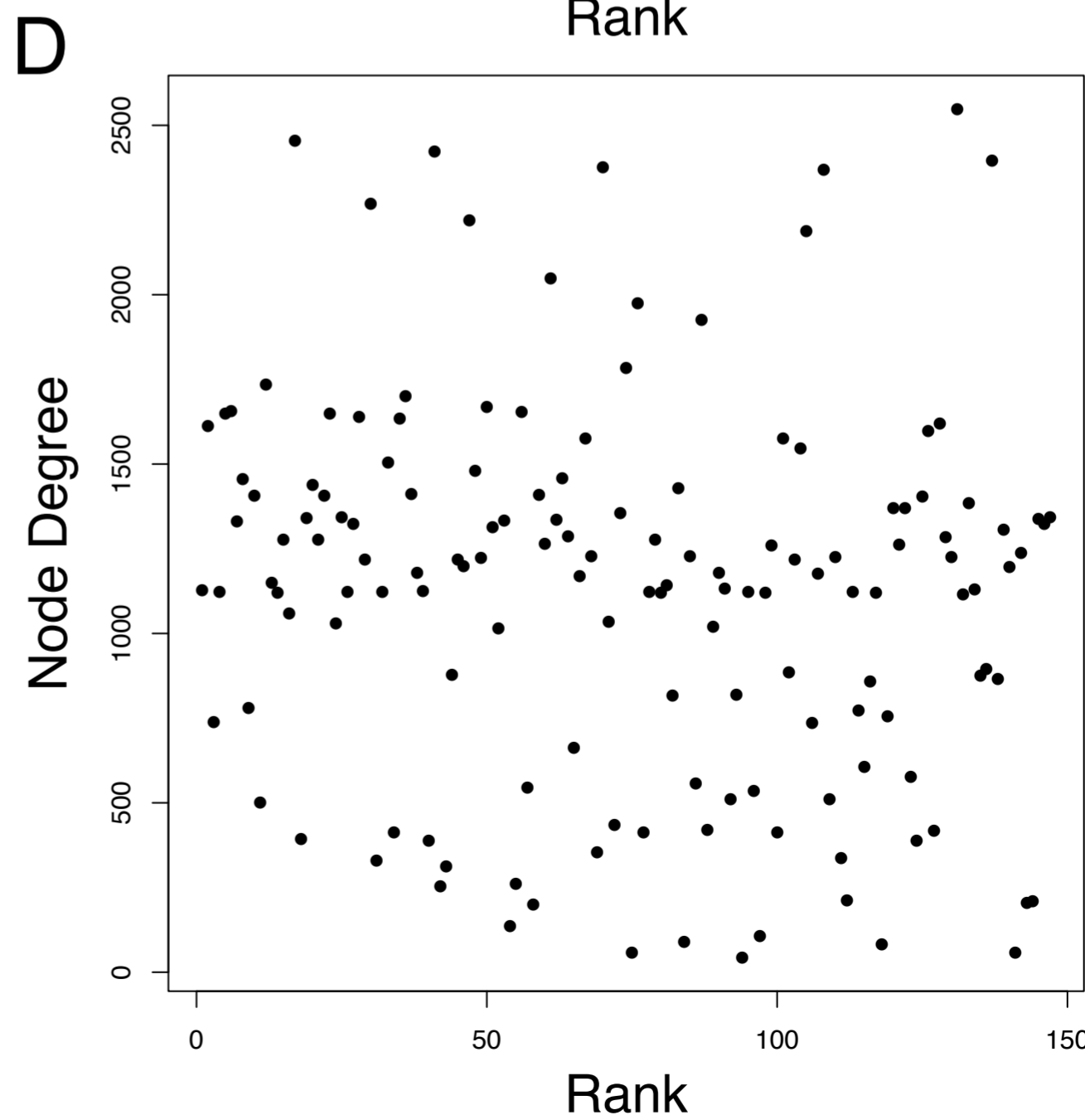

Supplement: Additional file 19 — Node degree in the influence graph versus the rank of significant genes (p ≤ 0.05) for the GBM (A), HGS (B), TN (C), and METABRIC (D) datasets. [file gb-2012-13-12-r124-S19.PDF]

Concordance with Cancer Gene Census

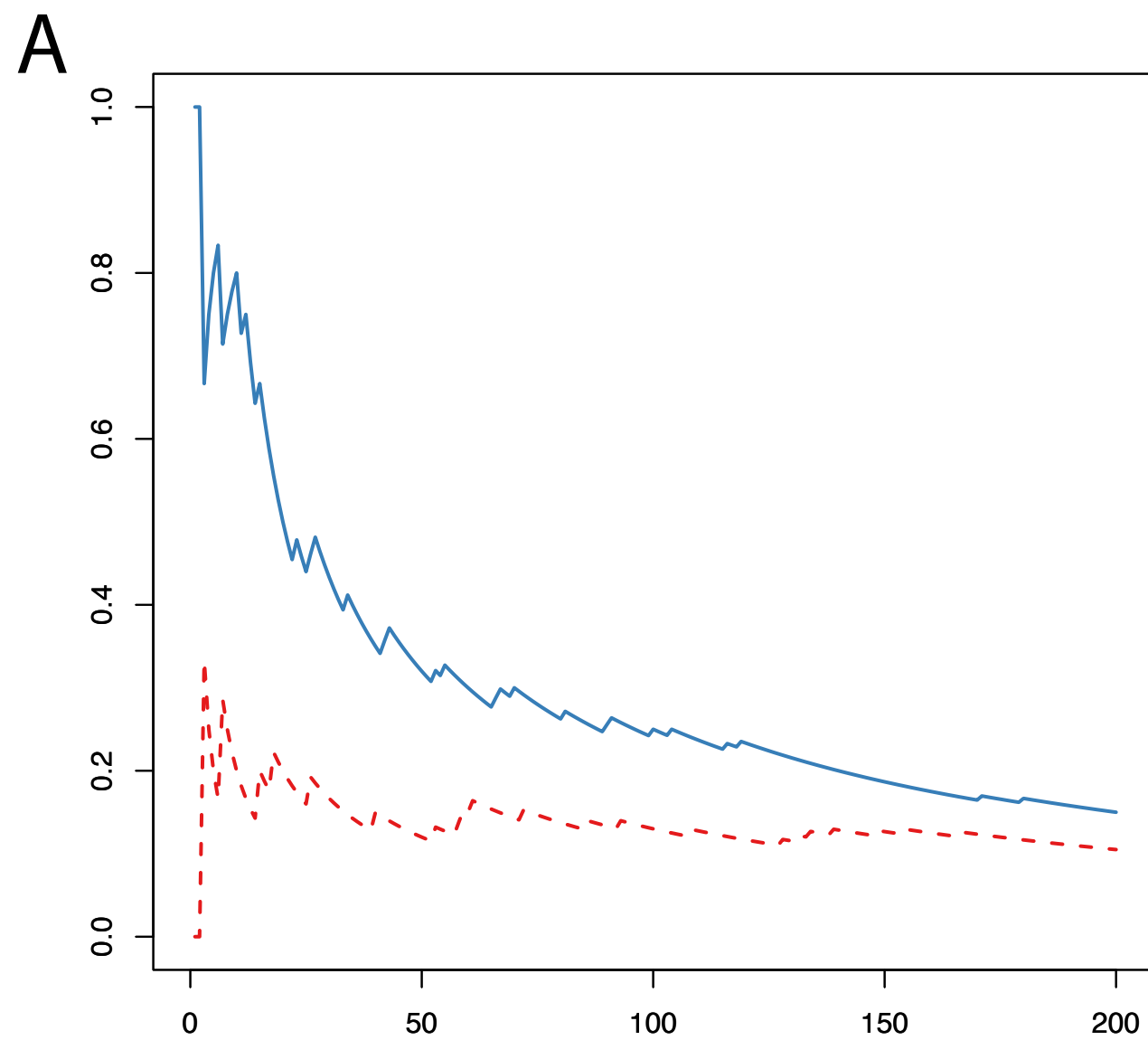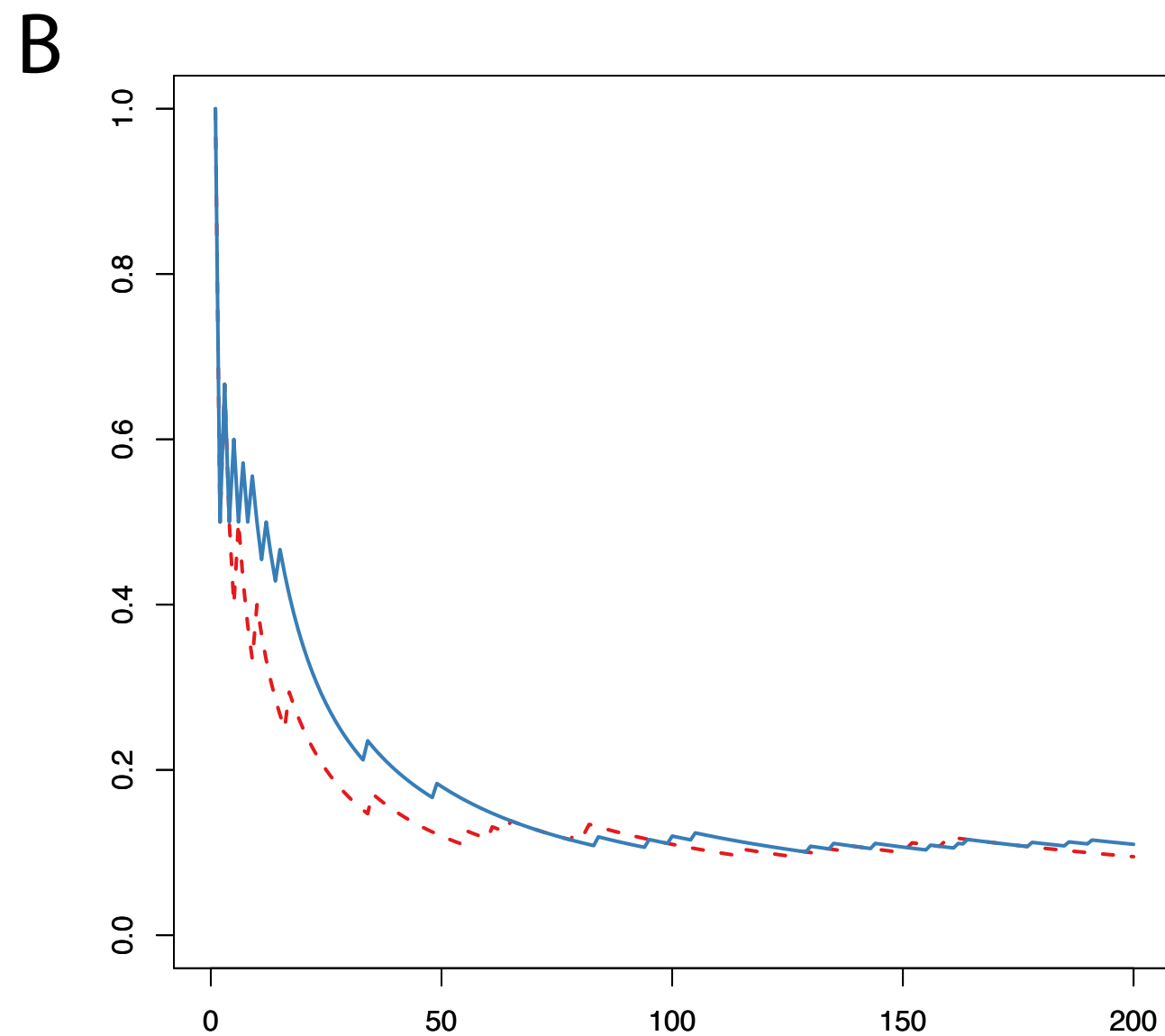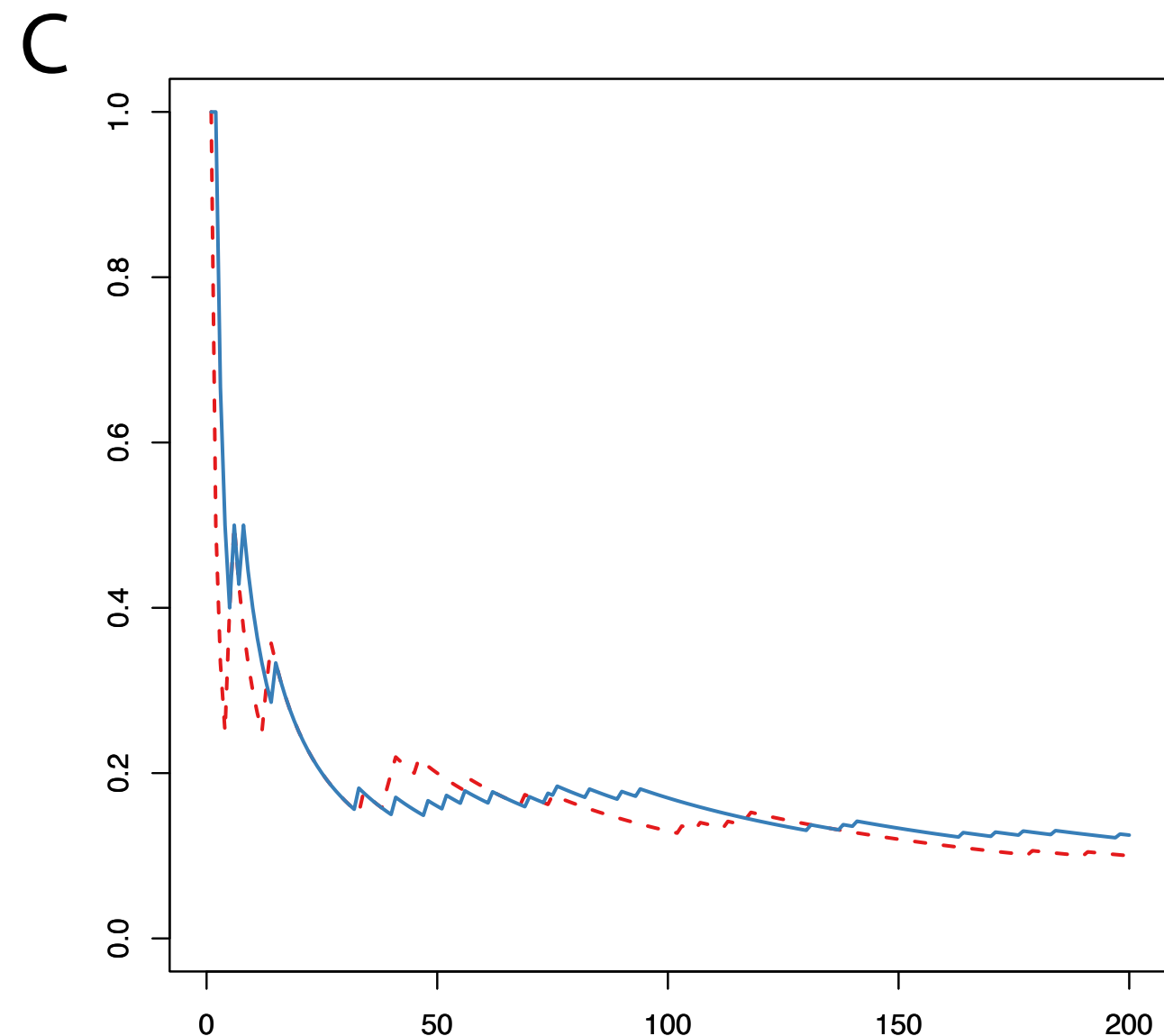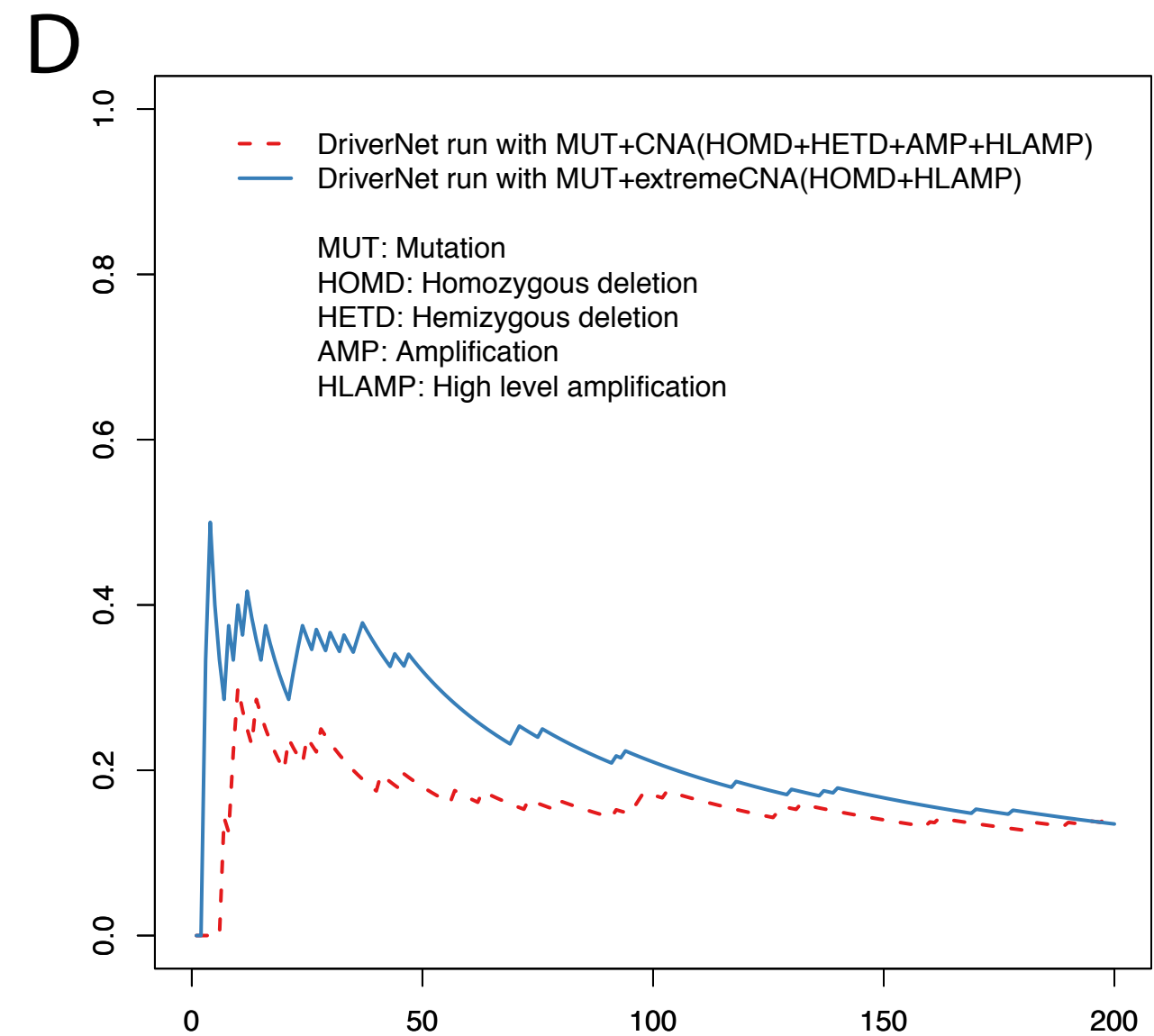

Concordance with COSMIC

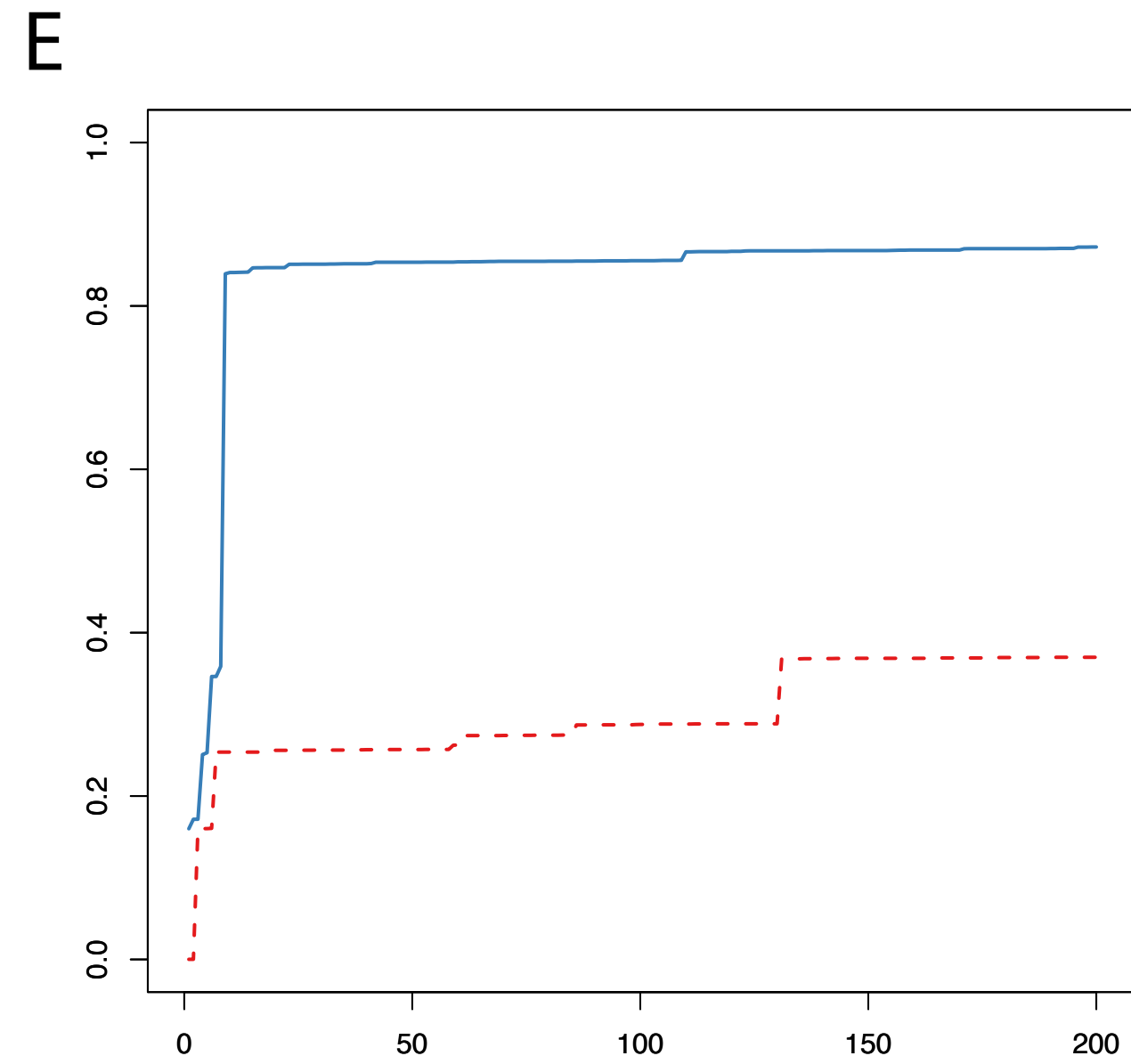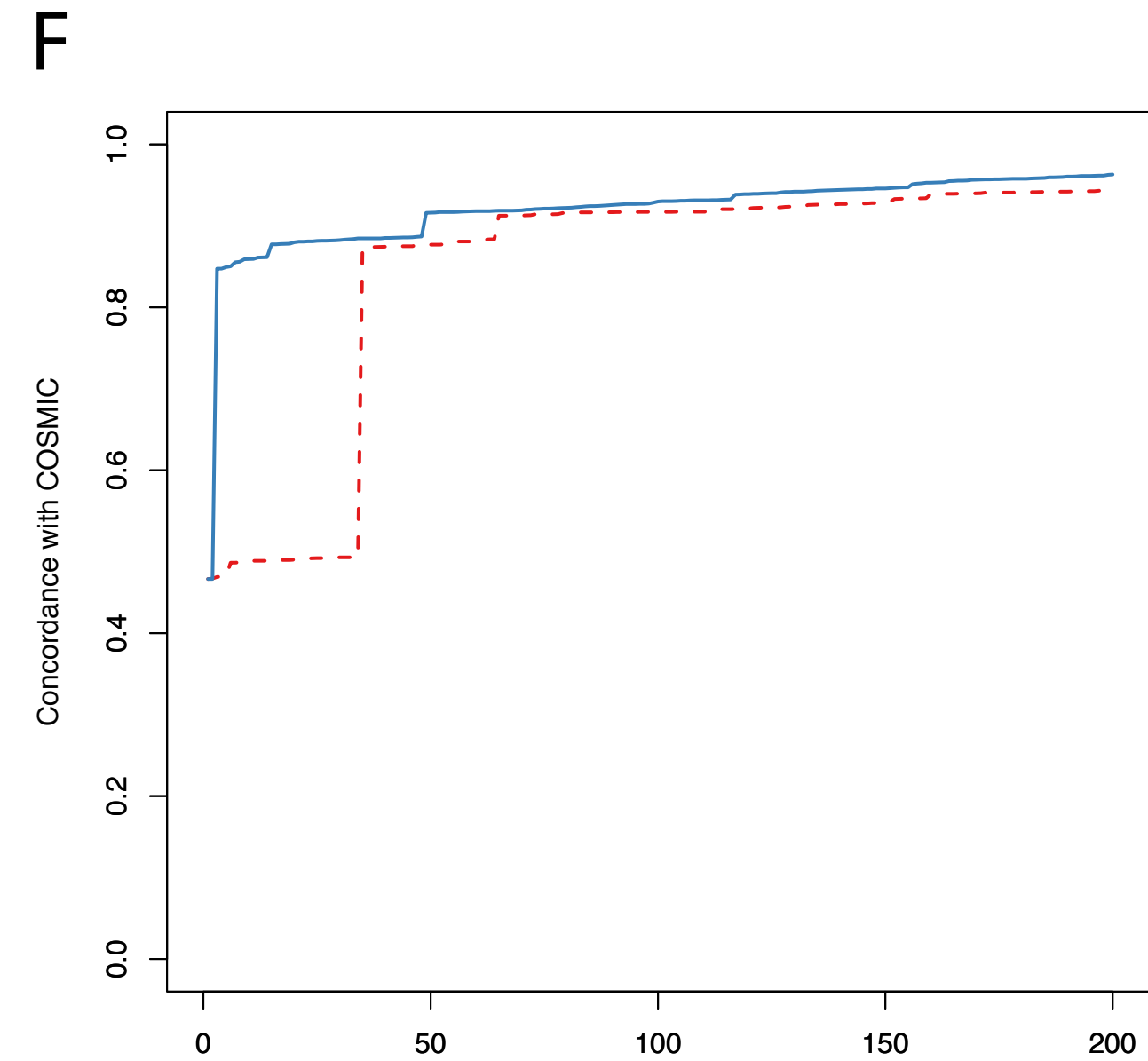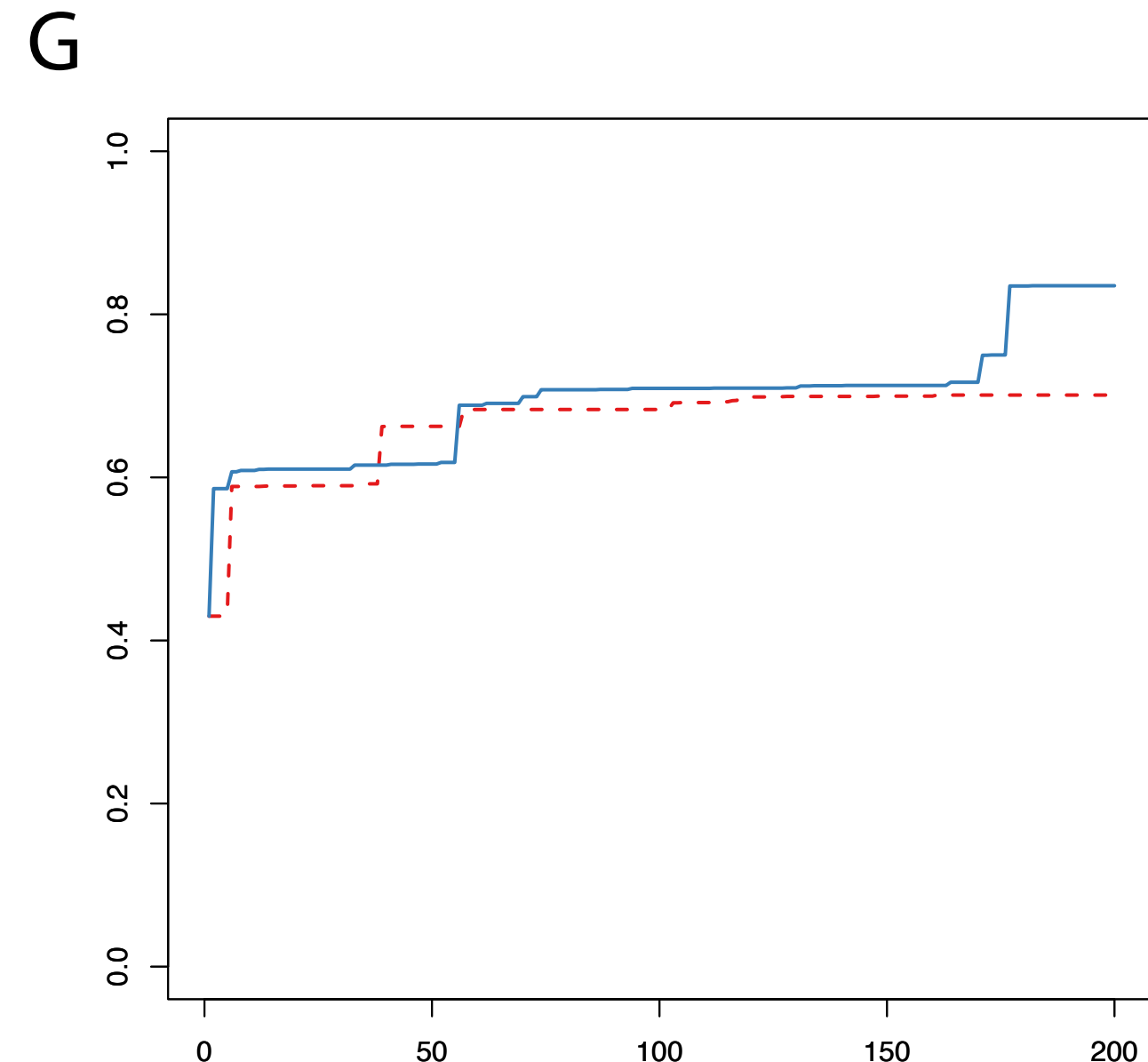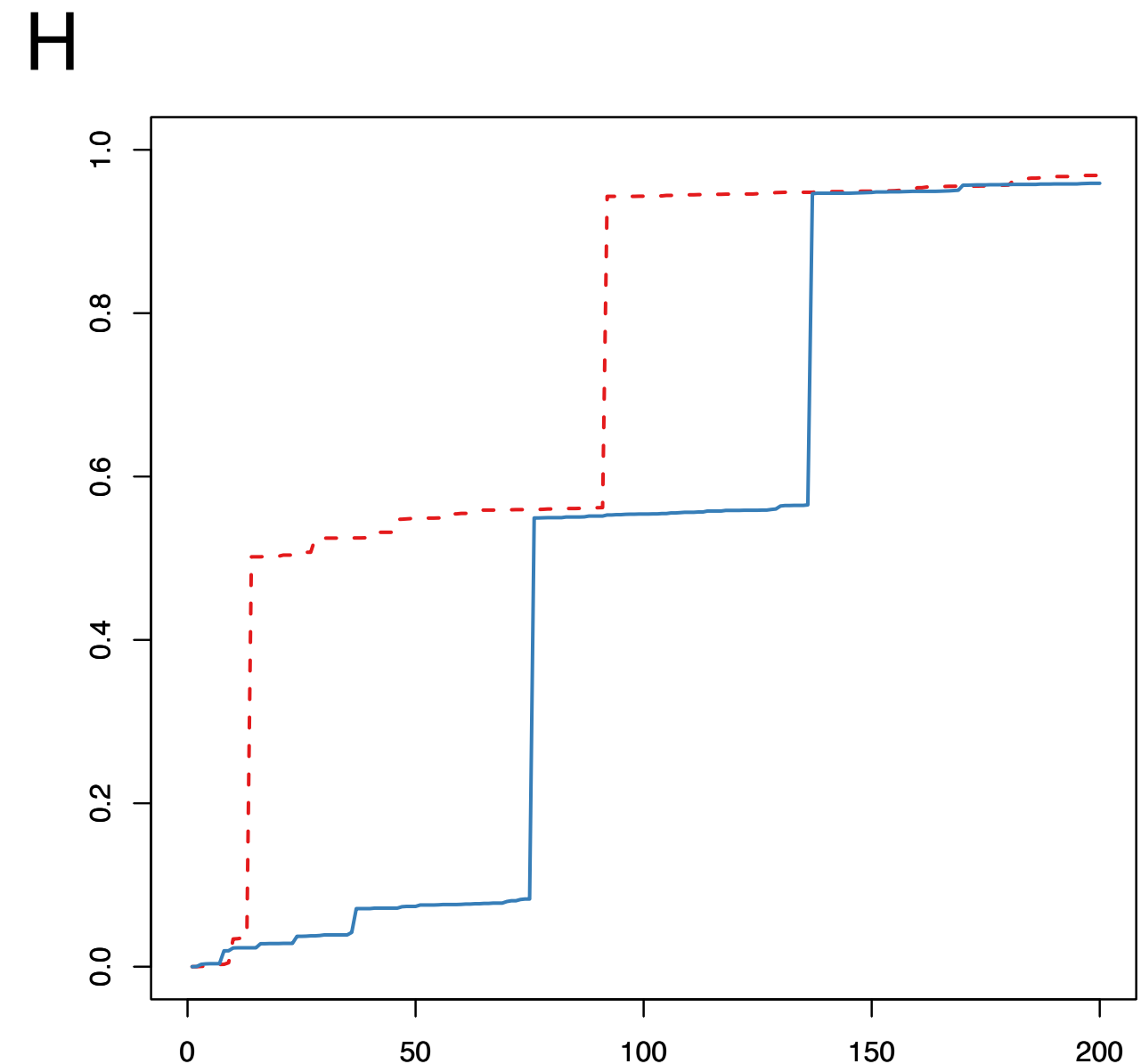

Supplement: Additional file 20 — DriverNet performance benchmarking on GBM, TN, HGS, and METABRIC datasets when copy number amplifications (AMP) and hemizygous deletions (HETDs) were included in addition to the high-level amplifications (HLAMP) and homozygous deletions (HOMDs). (A-D) Concordance with Cancer Gene Census for DriverNet, Frequency-based, and Fisher-based approaches as a function of the top N ranked genes (out of 200) for the GBM, TN, HGS, and METABRIC datasets, respectively. (E-H) Concordance with COSMIC database (cumulative distribution of mutation prevalence in the COSMIC database) for DriverNet, Frequency-based, and Fisher-based approaches as a function of the top N ranked genes (out of 200) for the GBM, TN, HGS, and METABRIC datasets, respectively. [file gb-2012-13-12-r124-S20.PDF]
