# Supplementary material for: DriverNet: uncovering the impact of somatic driver mutations on transcriptional networks in cancer
Source: Genome Biol. 2012 Dec 22;13(12):R124. doi: 10.1186/gb-2012-13-12-r124 (PMC4056374; doi:10.1186/gb-2012-13-12-r124)
Supplement: Additional file 17 — Circos plots showing outlying genes related to metabolic pathways for PNMT (A), NDUFC2 (B), and MTAP (C) and outlying genes related to oncogenic/tumor suppressor pathways for ERBB2 (D), PAK1 (E), and CDKN2A (F) genes. [file gb-2012-13-12-r124-S17.PDF]

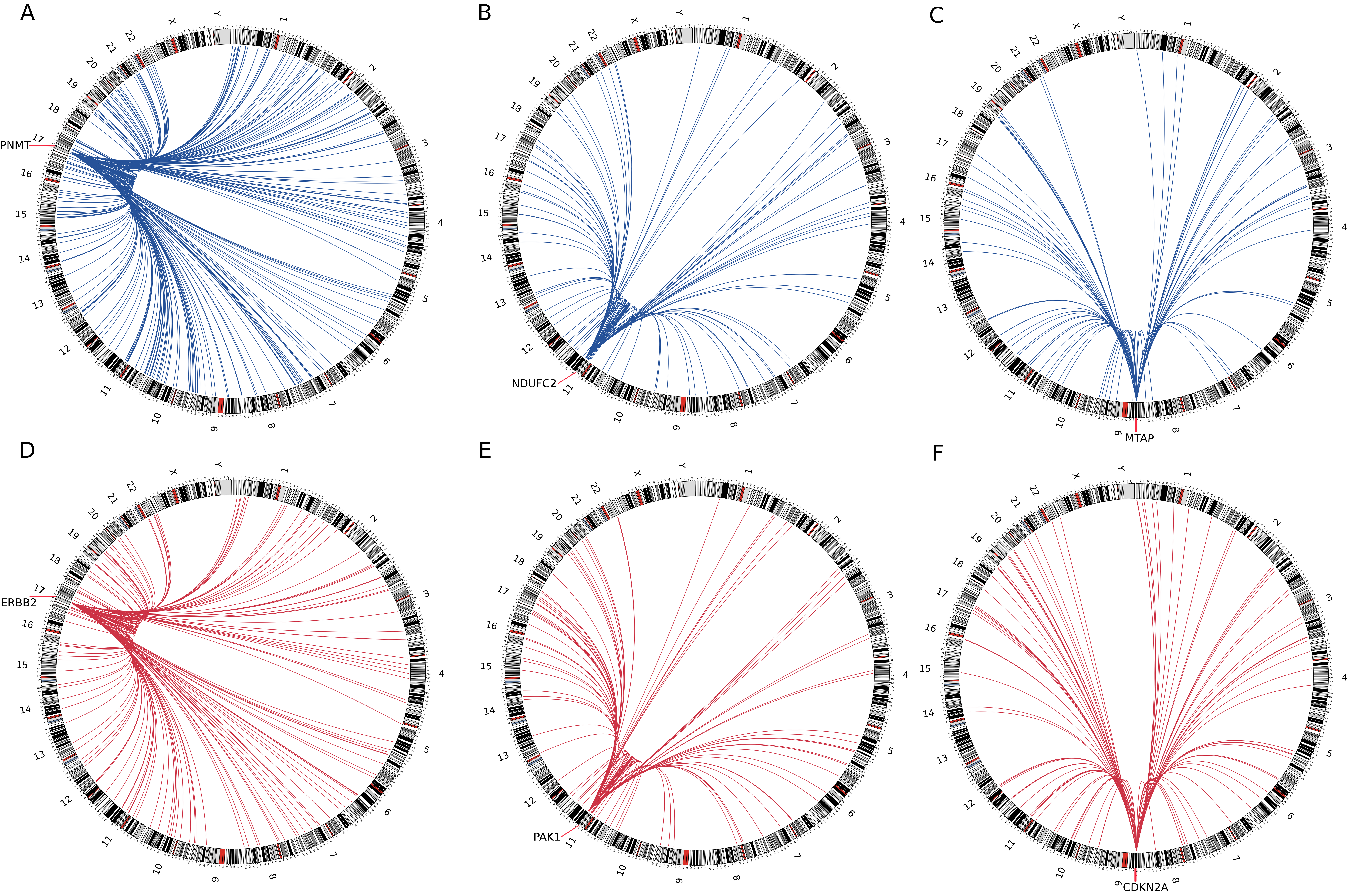
A
PNMT
B
NDUFC2
C
MTAP
D
ERBB2
E
PAK1
F
CDKN2A
1 2 3 4 5 6 7 8 9 10 11 12 13 14 15 16 17 18 19 20 21 22 X Y
